# Ruthenium-Based Photoactivated Chemotherapy

**DOI:** 10.1021/jacs.3c01135

**Published:** 2023-10-17

**Authors:** Sylvestre Bonnet

**Affiliations:** Leiden Institute of Chemistry, Leiden University, Einsteinweg 55, 2333CC Leiden, The Netherlands

## Abstract

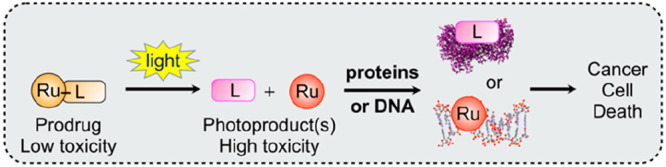

Ruthenium(II) polypyridyl complexes form a vast family of molecules characterized by their finely tuned photochemical and photophysical properties. Their ability to undergo excited-state deactivation via photosubstitution reactions makes them quite unique in inorganic photochemistry. As a consequence, they have been used, in general, for building dynamic molecular systems responsive to light but, more particularly, in the field of oncology, as prodrugs for a new cancer treatment modality called photoactivated chemotherapy (PACT). Indeed, the ability of a coordination bond to be selectively broken under visible light irradiation offers fascinating perspectives in oncology: it is possible to make poorly toxic agents in the dark that become activated toward cancer cell killing by simple visible light irradiation of the compound inside a tumor. In this Perspective, we review the most important concepts behind the PACT idea, the relationship between ruthenium compounds used for PACT and those used for a related phototherapeutic approach called photodynamic therapy (PDT), and we discuss important questions about real-life applications of PACT in the clinic. We conclude this Perspective with important challenges in the field and an outlook.

## 1 Phototherapies: An Introduction

### 1.1 Phototherapies in Medicine

Our eyes are not the only light-sensitive organs in humans. Our moods, our sleep, and our skin are also sensitive to sunlight. Artificial light sources entered clinical practice a long time ago, for example, to treat smallpox.^1^ Newborn jaundice treatment is one of the best-known clinical application of phototherapy,^2^ while skin tumors were treated with phototherapy in ancient Egypt.^3^ Inspired in part by naturally photoactive compounds,^4^ and driven by the development of antibiotic-resistant bacteria, new applications of phototherapy have developed rapidly, such as antibacterial photodynamic therapy (aPDT).^5^ However, the most developed application of medicinal phototherapy targets tumors.

### 1.2 Anticancer Phototherapies

Techniques to treat cancer patients using light-sensitive compounds have emerged to circumvent the toxicity of conventional treatments. In photodynamic therapy (PDT), a clinically approved treatment of pre-cancerous diseases of the skin or esophagus or of more advanced cancers of the brain or lungs, the photosensitive compound is called a “photosensitizer” (PS). Upon light excitation followed by spin flip, the PS is promoted into a triplet excited state (^3^PS*) that transfers an electron (PDT type I) or energy (PDT type II) to the O_2_ molecules present in the irradiated tissues. Such a transfer produces high local doses of reactive oxygen species (ROS) that generate three effects. First, they kill cancer cells by oxidative damage to nucleic acids, proteins, and lipid membranes. Second, they consume oxygen and damage blood vessels, thus generating hypoxia. Third, they trigger the immune system.^6^ Altogether, PDT often generates a strong antitumor effect with minimal side-effects for the patients. These factors explain, in part, the fast growth of clinical PDT and the number and quality of reviews dedicated to it.^7,8^

A second form of anticancer phototherapy involves organic protein inhibitors covalently functionalized with reversible photoswitches such as azobenzene or diarylethene.^9,10^ In azobenzene conjugates, the dark form of the prodrug has a *trans* azo bond, while its light-activated form is *cis*. Both forms show different interactions of the inhibitor with the target protein, which modulates protein activity and sometimes kills cancer cells upon light irradiation. With azo compounds, however, the *cis* form is thermally unstable and reverts to the more thermodynamically stable *trans* form, thereby leading to *reversible* prodrug light activation.

**Figure 1:**
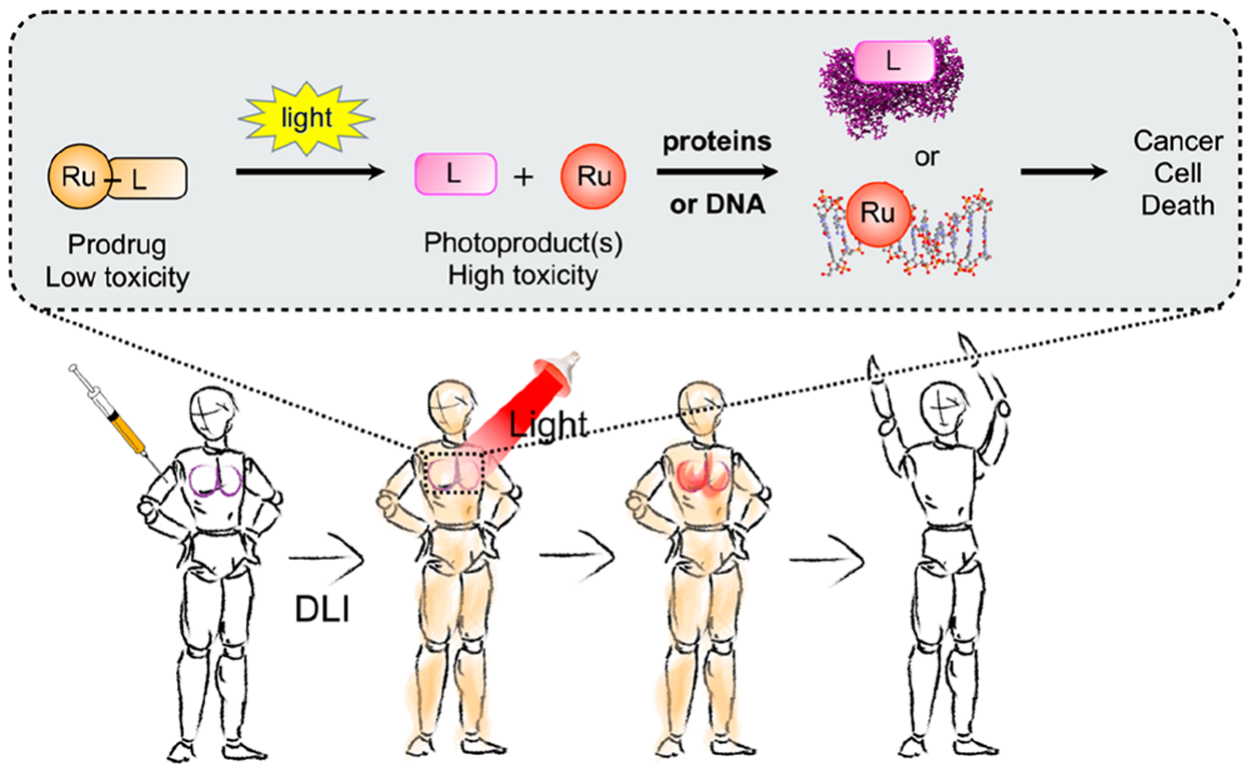
Principle of ruthenium-based photoactivated chemotherapy (PACT). Top: light-induced bond cleavage reaction in the prodrug. Either the photoreleased ligand (L) or the metal fragment (Ru), or both, interact(s) with biomolecules, leading to cell death. Bottom: PACT treatment of a patient with a lung tumor (in purple). The prodrug (orange) is injected intravenously, distributes in the body, and reaches the tumor in its non-toxic form. After the drug-to-light interval (DLI), light is shone onto the tumor, activating the prodrug and destroying the tumor. Finally, the body excretes the excess drug. Image courtesy Bianka Siewert.

The third main form of anticancer phototherapy, which is the focus of this Perspective, is called PhotoActivated ChemoTherapy (PACT). For clinicians, PACT may look like PDT (Figure 1): the patient receives a non-active prodrug, which distributes in the body and inside the tumor without causing harm. After some time, called the drug-to-light interval (DLI), light is shone onto the tumor, where it activates the prodrug. Finally, the activated compound and tumor debris are excreted outside the body. Chemically speaking, however, PACT addresses both the oxygen dependence of PDT and the reversibility of photoswitches in photopharmacology. It relies on the irreversible and oxygen-independent photochemical bond cleavage of either a metal–ligand coordination bond^11^ or a carbon–oxygen bond.^12^ In fact, PACT is similar to photocaging, a technique that uses a non-toxic “caging” group to “hide” the biological activity of a molecule, for example, ATP^13^ or morphin.^14^ When “caged”, the bioactive compound cannot interact with its target because the photocage ruins the precise key–lock fit built by medicinal chemists. Upon photochemical “uncaging”, the bioactive compound recovers its ability to interact with its biological target. Importantly, for PACT treatment of cancer, the prodrug in its photocaged form should be poorly toxic, while after light activation, at least one of the two photoreleased fragments should be very toxic to cancer cells. Inorganic photochemists have used different metal centers to prepare photocaged compounds that are activated with visible or near-infrared (NIR) light.^15,16^ Ruthenium-based photocages for the PACT treatment of cancer are the focus of this Perspective.

### 1.3 Early Developments of Metal-Based PACT Compounds

Historically, PACT is based on the concomitant development of PDT and platinum chemotherapy drugs. While Figge (1955) first detected tumor fluorescence upon hematoporphyrin injection,^18^ Dougherty shone light on hematoporphyrin-injected mice and patients (1975–1979) and demonstrated that singlet oxygen (^1^O_2_) was the cytotoxic agent.^19^ In parallel, cisplatin was discovered as a potent chemotherapeutic agent^20^ and was approved for clinical use in 1978. While Malik and Kennedy developed PDT using 5-aminolevulinic acid (1987–1990), Photofrin was approved by the FDA in 1995. The first article mentioning a “photo cisplatinum reagent”, from Morrison,^21^ dealt with the rhodium(III) polypyridyl complex [Rh(phen)_2_Cl_2_] (phen = 1,10-phenanthroline). This complex was able to photosubstitute one of its chloride ligand by a DNA base pair upon UV light irradiation.^22^ Though no biological experiments were initially performed, UV light irradiation was suggested to trigger metal coordination to DNA with light, which opened the door to photoinorganic therapeutic approaches.

The first platinum-based PACT prodrugs originated with Bednarski and Sadler.^23,24^ The activation mechanism for these thermally inert Pt(IV) compounds is different from that of rhodium(III) and ruthenium(II) polypyridyl complexes: upon light irradiation in cells, Pt(IV) compounds are photoreduced into a more labile platinum(II) photoproduct capable of exchanging ligands with biomolecules and finally binding to DNA. Finally, the first ruthenium(II) polypyridyl PACT compounds working by photosubstitution were proposed by Etchenique and Turro in 2003 and 2004, respectively.^25,69^ For Turro’s compound, the cytotoxic species was the metal-containing photoproduct, while in Etchenique’s case, the bioactive compound was the photosubstituted ligand. In parallel, thorough understanding of the photochemistry of ruthenium(II) polypyridyl complexes initiated by Sauvage,^26,27^ Balzani,^11,28^ McMillin,^29^ and Meyer^30^ led to fast developments of ruthenium-based PACT, leading to the first *in vivo* experiment by the Wu and Bonnet groups in 2016 and 2019, respectively. The term “photoactivated chemotherapy” (PACT) was proposed by Salder in 2009.^16^

## 2 Ruthenium-Based PACT Compounds

### 2.1 Photochemical Activation Mechanisms

**Figure 2:**
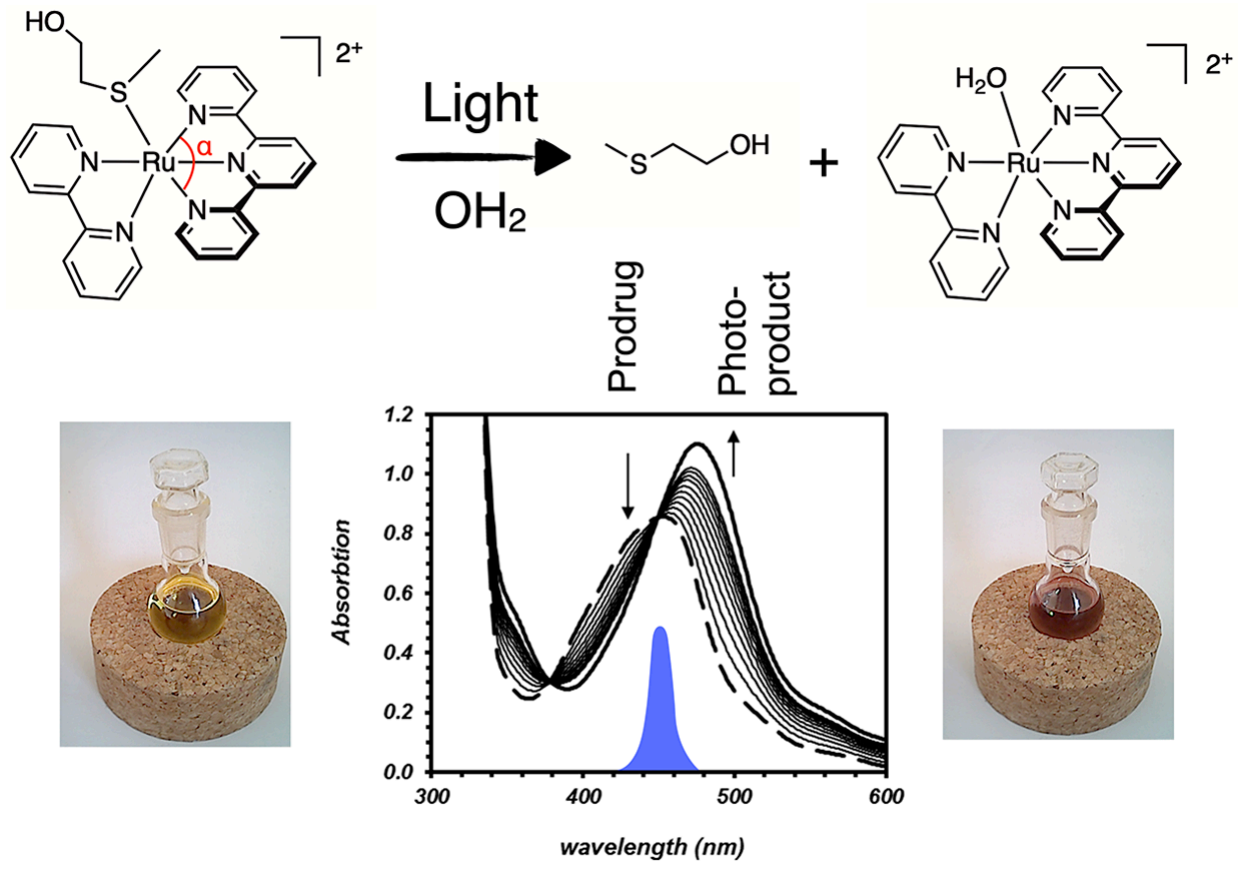
Example of a photosubstitution reaction used in PACT with ruthenium photocage [Ru(tpy)(bpy)(Hmte)]^2+^. The blue peak in the UV–vis spectrum shows the emission of the light source used to trigger photosubstitution, centered at 450 nm. The bottom graph shows the time evolution of the absorption spectrum of the solution during light irradiation. The low α angle (∼160°) in the terpyridine ligand distorts the first coordination sphere of the metal center compared to a perfect octahedron (180°), which facilitates photosubstitution. Data adapted from ref (17).

In ruthenium-based PACT compounds, a coordination bond between the ruthenium center and an organic ligand is broken via a photosubstitution reaction (Figure 2). In order to show this type of reactivity, the ruthenium center should be in the oxidation state +II and bound to a so-called “polypyridyl” chelate comprising at least two pyridyl rings connected to each other via a C–C bond. Both the 2,2′;6′,2″-terpyridine (tpy) and 2,2′-bipyridine (bpy) chelates in Figure 2 are typical examples of such polypyridyl ligands. Initially seen as a detrimental decomposition pathway for ruthenium-based photosensitizers in photocatalysis,^30^ photosubstitution reactions have since then proven to be useful tools for the controlled activation of molecular machines^31,32^ or anticancer drugs.^33^ In fact, photosubstitution reactions in ruthenium(II) polypyridyl complexes are rather unique, because they occur with good quantum yields upon irradiation with visible light, while the complexes are usually thermally inert. However, photosubstitution is not strictly reserved to Ru(+II) complexes: it has also been reported for low-spin polypyridyl d^6^ transition metal centers based on Ir(+III), Rh(+III), or Re(I) for example. Fe(+II) complexes are difficult to use for PACT because they are thermally labile, though photosubstitution with strong ligands (CO, CN^–^, or NO) has been described.^34^ Finally, photosubstitution on Os(+II) complexes is very rare and very slow.^35^

**Figure 3:**
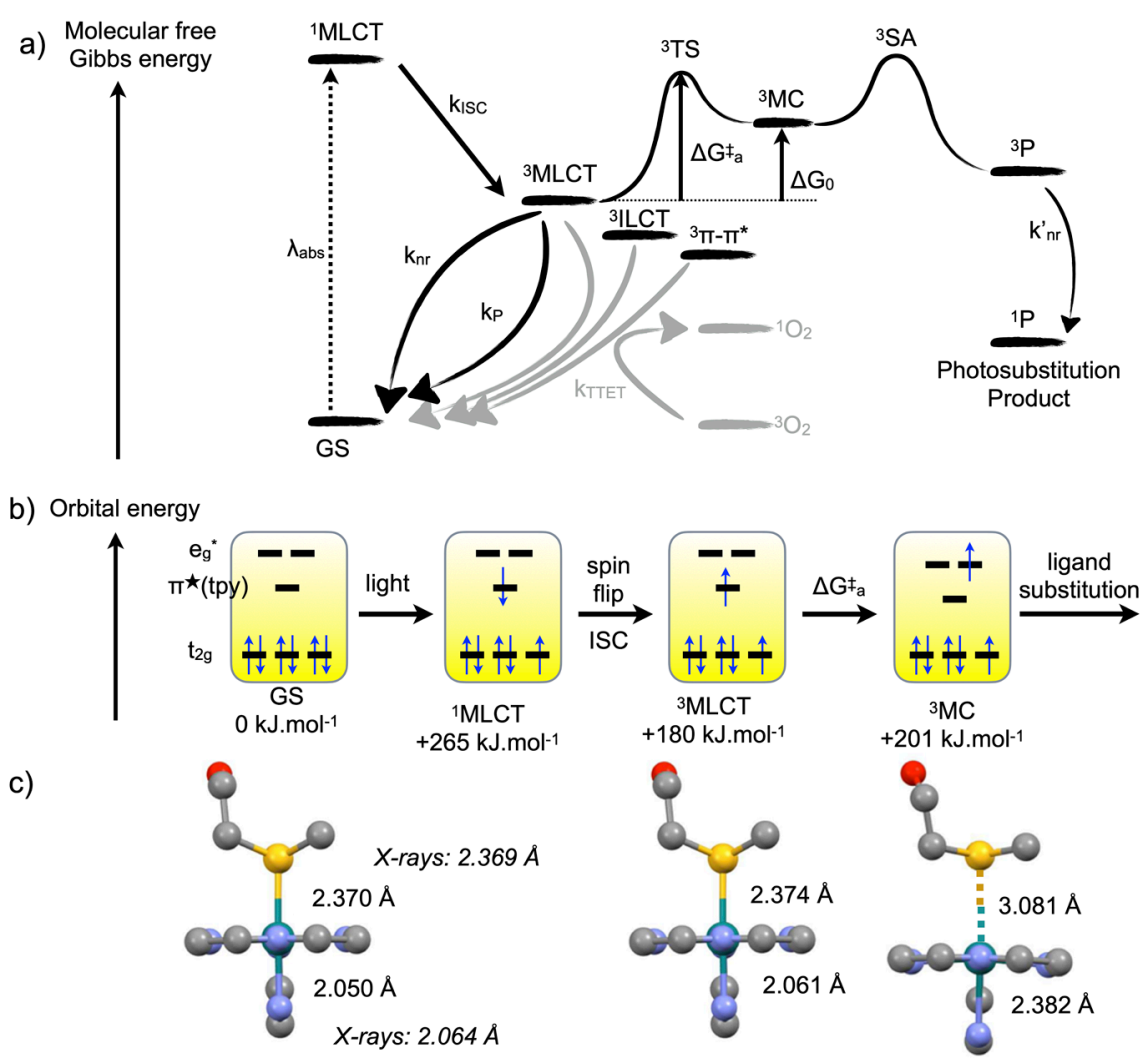
Classical model for photosubstitution reactions in ruthenium(II) polypyridyl complexes. (a) Molecular energy of the different states involved in the photochemistry of ruthenium(II) polypyridyl complexes. Gray pathways generate ^1^O_2_ in the presence of dioxygen; black pathways remain in the absence of O_2_. *k*_ISC_, *k*_nr_, *k*′_nr_, *k*_P_, and *k*_TTET_ are rate constants for intersystem crossing, non-radiative decay, phosphorescence, and triplet–triplet energy transfer, respectively. Δ*G*^⧧^_a_ is the activation barrier for the conversion of the ^3^MLCT to the ^3^MC state, and Δ*G*_0_ = *G*(^3^MC) – *G*(^3^MCLT). ^3^SA represents a Solvent Adduct of the complex in the triplet state. (b) Orbital energy scheme of the excited states involved in photosubstitution. Numerical values for bond lengths are indicated for [Ru(tpy)(bpy)(Hmte)]^2+^ (Figure 2), as reported in ref (50).

In polypyridyl ligands, conjugation leads to low-lying π* orbitals ending up as the lowest unoccupied molecular orbital (LUMO) of their ruthenium(II) complexes. Upon photon absorption, the octahedral complex promotes an electron from a metal-centered t_2g_ (HOMO) orbital into the ligand-centered LUMO, thereby generating a metal-to-ligand charge-transfer singlet excited state (^1^MLCT) that efficiently spin-flips to a triplet (^3^MLCT, Figure 3a,b). The classical mechanism of photosubstitution in polypyridyl ruthenium(II) compounds starts from these ^3^MLCT states. While they are typically responsible for the phosphorescence, electron-transfer, or energy-transfer processes observed with photoinert compounds such as [Ru(bpy)_3_]^2+^, they can also be thermally promoted to a metal-centered (^3^MC) triplet excited states that lies close enough in energy (Figure 3a). Usually, while ^1^MLCT-to-^3^MLCT transitions are very fast (<100 fs) and thermally non-activated, ^3^MLCT-to-^3^MC conversions take time and occur via an activation barrier.^36^ The corresponding triplet transition state (^3^TS) is represented in Figure 3a. This thermal barrier is due to the different geometries of the ^3^MLCT and ^3^MC states: in the ^3^MC state, the electron promoted in an antibonding (e_g_*) metal–ligand orbital elongates the Ru–ligand bond distance, compared to ^3^MLCT states (Figure 3b,c). Such a longer distance facilitates substitution of the ligand by a solvent molecule before decaying to the ground state of the photosubstituted product. This mechanism derives from ancient^37,38^ temperature-dependent phosphorescence lifetime and photosubstitution measurements^39^ and has been confirmed by multiple reports.^40−42,36^ In short, enhanced quenching of the phosphorescence of [Ru(bpy)_3_]^2+^ at high temperatures suggested that nearby ^3^MC states may be thermally populated from the photochemically generated ^3^MLCT states. From the temperature dependence of the phosphorescence lifetime and quantum yield, Watts and Houten derived an excess energy of Δ*G*_0_ ≈ +43 kJ/mol.^37^ This value was later confirmed by measuring the increase of photosubstitution quantum yield with temperature.^38^ Sauvage’s observation that more sterically hindered compounds showed more pronounced photosubstitution at the cost of phosphorescence^43^ confirmed the role played by ligand-field ^3^MC states in photosubstitution. It also demonstrated that ligand design can fine-tune the relative energies of the ^3^MC and ^3^MLCT states to favor photosubstitution.

As a note, different ^3^MC states may exist, characterized by different geometries and energies, in particular when different ligands may be photosubstituted.^44,36^ Recently, Elliott and Dixon suggested that, for tris-diimine complexes such as [Ru(bpy)_3_]^2+^, ^3^MC states reminiscent of *trans* bond activation may be responsible for non-radiative decay of the ^3^MLCT states (*k*_nr_ in Figure 3a), while photosubstitution may preferentially occur from *cis*^3^MC states.^45,46^*Cis* and *trans*^3^MC states are unrelated to *cis*- and *trans*-[Ru(bpy)_2_(OH_2_)_2_]^2+^ or [Ru(bpy)_2_(MeCN)_2_]^2+^ photoproducts, which may also interconvert upon prolonged light irradiation in water or acetonitrile, respectively, after initial photosubstitution of a bidentate ligand.^47−49^ Overall, the triplet hypersurface of ruthenium polypyridyl complexes is topologically complicated, and several ^3^MLCT, ^3^MC, intraligand charge transfer (^3^ILCT), or more localized (^3^π–π*) excited states may coexist and interchange upon light irradiation of a complex, thus leading to a wide range of photochemistries.

**Figure 4:**
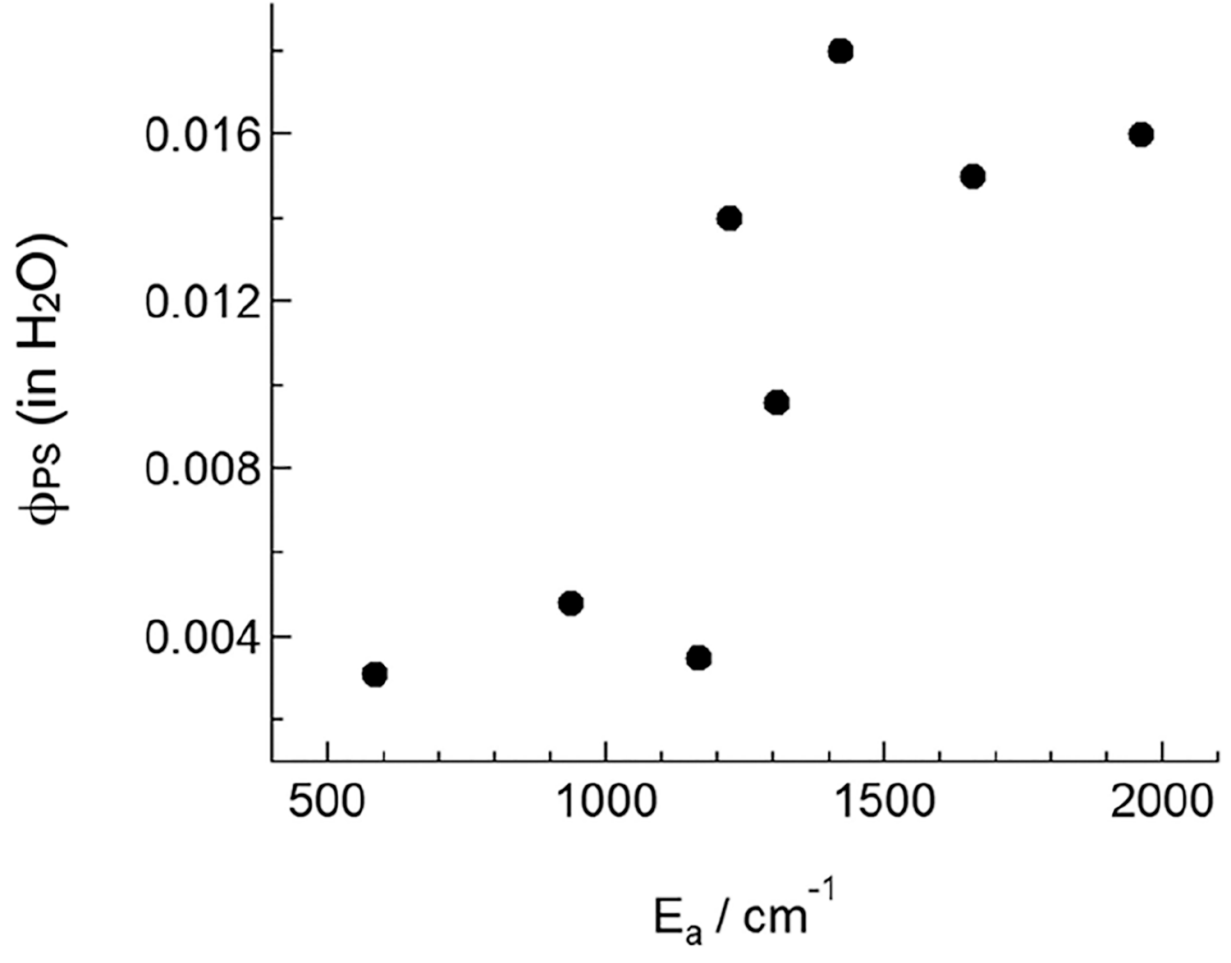
Relationship between the photosubstitution quantum yield in water (φ_PS_) and the activation energy (*E*_a_) to promote the ^3^MCLT state to the ^3^MC state, in [Ru(tpy)(L)(MeCN)]^*n*+^ (*n* = 1 or 2), where L is a bidentate ligand. Each dot represents a metal complex. Adapted from ref (54). Copyright 2022 American Chemical Society.

**Figure 5:**
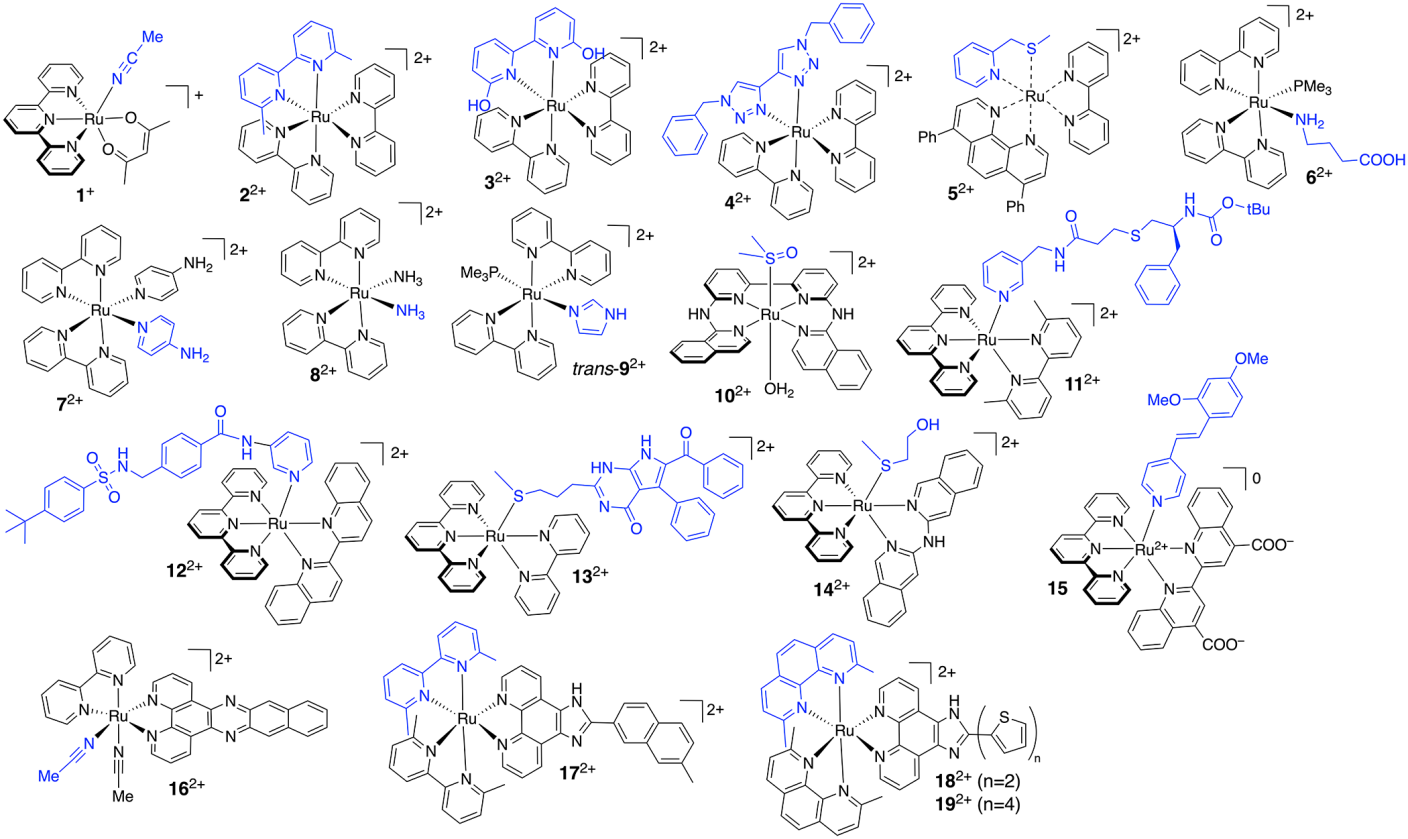
Selection of ruthenium-based PACT compounds. The first photosubstituted ligand is highlighted in blue.

Recently, the Turro group found that the photosubstitution of monodentate nitriles in [Ru(tpy)(acac)(RCN)]^+^ complexes (**1**^+^ for R = Me, acac^–^ = acetylacetonate, see Figure 5) was possible using far-red light (655 nm), while in [Ru(tpy)(bpy)(RCN)]^+^ complexes one should irradiate in the blue region (450 nm) to obtain photosubstitution.^51^ Further mechanistic studies demonstrated that photosubstitution in [Ru(tpy)(acac)(RCN)]^+^ did not follow the energy gap law.^52^ In other words, the ^3^MLCT lifetimes increased as its energy went down, thus making these states less prone to deactivate via ^3^MC states. In parallel, the complexes with the lowest ^3^MLCT had surprisingly the highest photosubstitution quantum yields. Thus, the acetylacetonate chelate, which is known to generate low-lying, poorly distorted MLCT states, led to longer ^3^MLCT lifetimes *and* higher photosubstitution quantum yields, while in the classical mechanism (Figure 3) lower photosubstitution quantum yields (φ_PS_) would be expected for low-lying ^3^MLCT states. This observation suggested that photosubstitution may also occur directly from the ^3^MLCT state, without thermal promotion to the ^3^MC. This striking observation was qualitatively confirmed by the Bonnet group in a series of [Ru(tpy)(N-N)(Hmte)]^2+^ (Hmte = 2-methylthioethanol) complexes^53^ and recently more directly demonstrated by Turro et al.^54^ As shown in Figure 4, the φ_PS_ values in Turro’s complexes increased when the activation barrier *E*_a_ (∼Δ*G*^⧧^_a_ in Figure 3) increased. There is currently no solid theoretical model that explains this recent observation. However, additional discussion on this topic can be found in a recent review.^33^

### 2.2 Molecular Design of Ruthenium-Based PACT Compounds

Despite these recent results, the current understanding of the photochemistry of ruthenium polypyridyl complexes is good, and several molecular design principles for PACT compounds have been established (Figure 5). The molecular structure influences both the photosubstitution mechanism and they quantum efficiency, but also the light absorption properties of the complex. One of the most studied families of ruthenium complexes investigated for PACT is based on complexes bound to three diimine chelates.^55−57^ The reference compound, [Ru(bpy)_3_]^2+^, is weakly phosphorescent (φ_P_ ≈ 0.02) and a good generator of ^1^O_2_ (φ_Δ_ ≈ 0.73), but it is not a PACT compound: The *cis*^3^MC states are high in energy, which prevents photosubstitution at body temperatures. Notably, bpy photosubstitution does occur in near-boiling (90 °C) HCl aqueous solutions because Δ*G*^⧧^_a_ is not infinite.^38^ The Sauvage group first reported that introducing steric hindrance in such complexes triggered photosubstitution at room temperature.^43^ Inspired by this approach, the Glazer group has developed, since 2012,^55^ a series of sterically hindered tris-diimine complexes for PACT.^58,59^ In those compounds (**2**^2+^, Figure 5), steric hindrance comes from the methyl groups *ortho* to the nitrogen bpy (or phen) atoms. For example, the sterically hindering 6,6′-dimethyl-2,2′-bipyridine chelate (dmbpy) introduces distortion of the first coordination sphere of the complex, which lowers the ^3^MC energy and triggers efficient photosubstitution. The Papish group developed analogous complexes (**3**^2+^, Figure 5) based on 6,6′-dihydroxy-2,2′-bipyridine (dOHbpy). **3**^2+^ photosubstitutes dOHbpy in acidic conditions (pH 5.0) where phenols are protonated. At pH 7.5, however, the phenol groups become deprotonated, leading to a major shift of the photochemistry of the complex that becomes photostable and a good PDT sensitizer (Table 1).^56,60^

**Table 1:** Photochemical Properties of Selected Ruthenium(II) Polypyridyl Complexes Used in PACT^a^

	λ_max_^MLCT^	φ_PS_ (λ_exc_ in nm)^b^	φ_P_^c^	φ_Δ_^d^	ref
[Ru(bpy)_3_]^2+^	450	0 (450)^f^	0.015	0.73^i^	(66)
		0.00053 (436)^e^			(38)
[Ru(bpy)_2_(dmbpy)]^2+^ (**2**^2+^)	450	0.05 (413)^f^	0.00003	0.023^i^	(67)
[Ru(dpp)(bpy)(mtmp)]^2+^ (**5**^2+^)	430	0.111 (521)^f^	n.d.	0.03^i^	(64)
[Ru(phpy)(bpy)(mtep)^+^ (**23**^+^)	526	0.00035 (521)^f^	n.d.	n.d.	(68)
[Ru(bpy)_2_(6,6′-dOHbpy)]^2+^ (**3**^2+^)	462	0.0058 (450)^g^	n.d.	0.041^i^	(60)
	493	0.0012 (450)^h^	n.d.	0.18^i^	(60)
[Ru(bpy)_2_(NH_3_)_2_]^2+^ (**8**^2+^)	490	0.024 (350)^j^	0.002	n.d.	(69)
[Ru(bpy)_2_(MeCN)_2_]^2+^	427	0.21 (40098)^j^	n.d.	n.d.	(70)
		0.22 (450)^j^			
[Ru(bpy)(dppn)(MeCN)_2_]^2+^ (**17**^2+^)	430	0.002 (400)^j^	n.d.	0.72^i^	(71)
*cis*-[Ru(bpy)_2_(PMe_3_)(ImH)]^2+^ (*cis*-**9**^2+^)	432	0.10^j^	n.d.	n.d.	(72)
*trans*-[Ru(bpy)_2_(PMe_3_)(ImH)]^2+^ (*trans*-**9**^2+^)	464	0.23^j^	n.d.	n.d.	(72)
[Ru(bapbpy)(dmso)(OH_2_)]^+^ (**10**^2+^)	308	0.003 (450)^j^	n.d.	0.013^i^	(73)
[Ru(tpy)(bpy)(dmso)]^2+^	411	0.016 (450)^f^			(74)
[Ru(phbpy)(bpy)(dmso)]^+^ (**22**^+^)	476	0.000041 (450)^f^	0.00016	0.032^i^	(74)
[Ru(tpy)(bpy)(Hmte)]^2+^	450	0.022 (452)^j^	<10^–4^	<0.005^i^	(75)
[Ru(tpy)(bpy)(R-SCH_3_)]^2+^ (**13**^2+^)	454	0.0038 (530)^f^	n.d.	n.d.	(76)
		0.0055 (450)			
[Ru(tpy)(dppn)(R-SCH_3_)]^2+^	458	0.00095 (450)^f^^j^	0.000037	0.71^i^	(77)
[Ru(tpy)(biq)(R-py)]^2+^ (**12**^2+^)	531	0.013 (625)^j^	n.d.	0.0036^i^	(78)
[Ru(tpy)(acac)(MeCN)]^+^ (**1**^+^)	505	0.014 (450)^j^	n.d.	n.d.	(51, 52)
[Ru(tpy)(bca)(R-py)]^0^ (**15**)	550	0.0081 (470)^k^	n.d.	n.d.	(79)
[Ru(tpy)(dmbpy)(R-py)]^2+^ (**11**^2+^)	474	0.15 (500)^j^	n.d.	n.d.	(80)
		0.31 (500)^f^			(80)

a In the chemical formulas, R represents different substituents (see original publications); the ligand abbreviations are indicated in the main text.

b Quantum yield for photosubstitution measured at the indicated excitation wavelength λ_exc_ and, unless otherwise noted, at room temperature.

c Phosphorescence quantum yield.

d Singlet oxygen (^1^O_2_) generation quantum yield. n.d. = not determined.

e Measured at 363 K in 0.1 M aqueous HCl.

f In acetonitrile.

g In aqueous solution at pH 5.0.

h In aqueous solution at pH 7.5.

i In CD_3_OD solution.

j In water.

k In H_2_O containing 5% DMSO.

Next to steric hindrance, electronic effects in tris-diimine ruthenium complexes may also trigger photosubstitution. The Elliott group developed a series of analogues of **2**^2+^ where the dmbpy chelate was replaced by a bis-triazole derivative (**4**^2+^, Figure 5).^61^ These ligands destabilize ^3^MLCT states rather than lowering ^3^MC states, which accelerates photosubstitution. Following studies from Jouvenot et al.,^62^ the Bonnet group replaced dmbpy by thioether-containing bidentate chelates such as 2-(methylthiomethyl)pyridine (mtmp)^63,64^ (**5**^2+^, Figure 5) or 1,3-bis(methylthio)-2-propane, which also led to efficient photosubstitution.^47^ Turro suggested^65^ that greater photosubstitution quantum yields were obtained with bis-thioethers due to the longer Ru–S bond distance elongation in the lowest triplet-state geometry. On the other hand, there was no indication about the nature (^3^MLCT vs ^3^MC) of these lowest triplet states. As pyridyl–thioether chelates have not yet been included in detailed theoretical studies, it is unclear at that stage why they lead to such good photosubstitution quantum yields. However, it is clear that they form excellent caging groups for ruthenium-based PACT compounds.^64^

A second family of ruthenium-based PACT compounds consists of complexes containing two *cis* monodentate ligands. Initially introduced by Etchenique,^25^ [Ru(bpy)_2_(L)(L′)]^2+^ compounds may release either one or two monodentate ligand(s), L and L′, depending on their chemical nature and on irradiation times. For example, monodentate phosphines (L = PPh_3_ or PMe_3_, see **6**^2+^ in Figure 5) are usually photostable, but they allow efficient photorelease of monodentate amines, pyridines, or nitriles (L′). Alternatively, two identical pyridines, primary amines or imidazoles (L = L′, **7**^2+^ and **8**^2+^Figure 5), may be photosubstituted successively. The second photosubstitution is much slower than the first one due to excited-state deactivation in the monoaqua intermediate [Ru(bpy)_2_(OH_2_)(L′)]^2+^. These compounds have been initially introduced for the photocaging of neurotransmitters (4-aminopyridine in **7**^2+^, γ-aminobutyric acid in **6**^2+^)^25,81−83^ but later on served as phototoxic warheads,^84^ as suggested by Turro.^69^ These compounds also exist in a *trans* form. Though less information is available on *trans* isomers, recently *trans-*[Ru(bpy)_2_(PMe_3_)(ImH)]^2+^ (*trans-***9**^2+^, ImH = imidazole, Figure 5) was shown to have red-shifted absorption, compared to its *cis* analogue, and also higher photosubstitution quantum yields (Table 1).^72^ This observation opens new design opportunities toward ruthenium-based PACT compounds with red-shifted activation.

Recently, the Bonnet group introduced a new family of tetrapyridyl complexes that, upon coordinating the basal plane of ruthenium, leave two *trans* coordination sites (**10**^2+^, Figure 5).^73^ In these compounds, light irradiation led to photosubstitution of the axial dmso ligand, which, when performed in cancer cells, led to cell death. The Glazer group recently demonstrated that *trans* ruthenium polypyridyl complexes may have improved toxicity compared to *cis* analogues, unlike for platinum compounds, for which transplatin is less active than cisplatin.^85^ Clearly, for ruthenium polypyridyl complexes, the relationship between the poorly toxic *cis* photocages and their more toxic *trans* analogues needs to be further investigated.

Another important family of ruthenium polypyridyl compounds used in PACT contains molecules based on tpy ligands (Figure 2).^86,87^ This scaffold generates N–Ru–N angles between N atoms of the terminal pyridyl rings of tpy that are much lower (α ≈ 150–160° in Figure 2) than the 180° angle expected in a perfect coordination octahedron. This low angle represents a significant distortion of the first coordination sphere of the metal, which significantly lowers the ^3^MC energy, thus shifting photoreactivity toward photosubstitution.^88^ A prototypic example is the [Ru(tpy)(bpy)(OH_2_)]^2+^ complex, which is poorly toxic by itself^89^ but binds to many monodentate ligands L that can thereafter be photosubstituted with good quantum yields (Figures 2 and 3b).^50,90^ This scaffold forms an excellent photocaging group, and a wide range of photocaged complexes of the type [Ru(tpy)(N-N)(L)]^2+^ have been published with different cytotoxic organic inhibitors L, and different bidentate spectator ligands N-N, such as dmbpy, 2,2′-biquinoline (biq), di(isoquinolin-3-yl)amine (i-Hdiqa), or bicinchoninic acid (H_2_bca), some of which (**11**^2+^–**14**^2+^, **15**) are shown in Figure 5 and Table 1. Four types of monodentate ligands L were considered for these photocages: thioether, nitriles, pyridines, and pyrazines.^15^ Importantly, the steric hindrance of the chelate N-N must be adjusted to the steric requirements of the monodentate ligand L. With L = thioethers, for example, the CH_2_ or CH_3_ substituents on sulfur come close to ruthenium upon coordination, which requires limited steric hindrance on the bidentate chelate: N-N should be an unsubstituted bipyridine (**13**^2+^) to keep good thermal stability.^17^ i-Hdiqa (**14**^2+^) provides higher steric hindrance and photosubstitution quantum yields without jeopardizing thermal stability (Table 1),^53^ but biq is too sterically hindered, resulting in a Ru–S bond that is thermally unstable in water.^17^ By contrast, monodentate L = pyridine ligands make thermally and photochemically non-labile complexes when N-N = bpy. To obtain efficient pyridine photolabilization, steric hindrance on the bidentate chelate N-N is required. The N-N = dmbpy, biq, and bca^2–^ chelates have allowed the caging of a wide series of pyridine-based inhibitors (**11**^2+^, **12**^2+^, or **15**, Figure 5).^78,80,79^ The advantage of the [Ru(tpy)(N-N)(L)]^2+^ scaffold is its great versatility and tunability. On the other hand, most of these complexes are activated by blue or green light, and only a small subset is sensitive to red light (usually 630 nm, but **15** is sensitive to 660 nm).^78^ Replacing the N-N chelate by an oxygen-based, monoanionic acetylacetonate ligand (**1**^+^) recently made it possible to shift the activation wavelength to the NIR region of the spectrum.^51^ The biology of these compounds has not been extensively evaluated, but apparently **1**^+^ is quite toxic in the dark.^91^ The Sun group recently made use of a similar ruthenium cage for activating tumor-targeted nanoparticles at 760 nm via a combination of PDT and PACT.^92^ This work demonstrates the high potential of [Ru(tpy)(O-O)(L)]^2+^ for anticancer phototherapy.

Finally, next to changing the first coordination sphere of ruthenium by fine-tuning the denticity, steric hindrance, and electronic effects of the ligands, PACT compounds can be functionalized on one of the “spectator” ligands by π-extended functional groups (**16**^2+^–**19**^2+^, Figure 5). Extended π substituents can have a profound influence on the photobiology of ruthenium polypyridyl complexes, and notably on their behavior in hypoxic cancer cells. In many cases, the extended π substituents introduce localized (^3^π–π*, **16**^2+^) or intraligand charge-transfer (^3^ILCT, **17**^2+^–**19**^2+^) excited states (Figure 3a) that generate new pathways for ^1^O_2_ or radical generation via energy or electron transfer, respectively. These effects were recently discussed in papers from Glazer and McFarland describing sterically hindered ruthenium complexes functionalized with naphthalene (**17**^2+^)^93,59^ or oligothiophene (**18**^2+^, **19**^2+^)^94^ groups, respectively. As first highlighted by Turro with **16**^2+^,^71^ extended π ligands often result in mixed photoreactivity combining PACT and PDT mechanisms. The photoindex values in so-called “dual action” phototherapeutic ruthenium compounds can be extremely high (up to 10^3^–10^6^ in hypoxic SKMEL28 cells^94^), which represents one of the great advances in the field of ruthenium-based anticancer phototherapy in the past few years.

### 2.3 About Photosubstitution Quantum Yields and Irradiation Times

One central question in PACT is what the photosubstitution quantum yield of a good compound should be. Photochemists are often used to compounds with ^1^O_2_ quantum yields or fluorescence quantum yields that are close to 1. However, in PACT, a photosubstitution quantum yield of 1 would be detrimental to preclinical developments, as photosubstitution is a decomposition reaction that changes the chemical structure of the molecule. A near-unity photosubstitution quantum yield means that each absorbed photon activates the molecule. In practice, chemists, biologists, or doctors studying such compounds would need to work in absolute darkness. In addition, tuning complexes toward higher photosubstitution quantum yields often lowers their dark stability, which lowers their photoindexes. As an example, compounds based on the [Ru(tpy)(dmbpy)(R-py)]^2+^ scaffold have been proposed that have photosubstitution quantum yields above 0.10; in our hands, the thermal stability of such compounds is insufficient.^78^ Overall, the most useful PACT compounds have photosubstitution quantum efficiencies of a few percent (0.01 to 0.10, see Table 1), while a few well-studied compounds have even lower φ_PS_ values (down to 0.001, see Table 1). Such quantum yields are excellent for activation *in vitro* or *in vivo* because LEDs and lasers are cheap and powerful: it is always possible to increase the number of photons shone onto a tissue and hence to activate a compound with a low φ_PS_ value. On the other hand, a photosubstitution quantum yield of a few percent is low enough to allow chemists to isolate compounds and study their biology in low-light conditions by protecting flasks, samples, or 96-well plates with opaque foil and brown glassware or Eppendorf.

In fact, the real question is how high the irradiation time and power density can go when performing *in vitro* and *in vivo* PACT experiments. *In vitro* irradiation of living cancer cells is typically performed using LED arrays placed above or below 96-well plates.^95^ It is difficult to irradiate such a plate for more than 60–90 min, as in most setups cells are deprived of CO_2_ and controlled humidity during light irradiation. As typical light intensities of LED devices are 10–50 mW/cm^2^, maximum light doses *in vitro* lie around 300 J/cm^2^. With such light doses, compounds with a quantum yield of 0.01 or more are perfectly activated *in vitro*,^73^ while quantum yields of 0.001 may start posing a problem. It should be noted that divergent beams from extremely powerful lasers (e.g., 5–15 W) can also be used to irradiate 96-well plates. Such setups allow for reaching much higher light intensities *in vitro* (e.g., 700 mW/cm^2^), which may be used to activate compounds with low photosubstitution quantum yields.^96^

*In vivo*, the irradiation time depends on the animal model. In mice or rats, the animal should not move during irradiation, so in most PDT or PACT studies it is anesthetized during light irradiation. The maximum irradiation time is hence determined by the maximum time allowed by ethical committees to keep an animal anesthetized, which is typically 15–20 min. We use typical values of fluence rate (or light intensity) of 50–150 mW/cm^2^*in vivo*; the maximum laser intensity allowed for medicine depends on the wavelength and organ irradiated, but for the skin intensities typical values of 0.7 W/cm^2^ are acceptable for visible light. The corresponding light doses (or fluence values) would be typically 50–100 J/cm^2^, and the maximal fluence values would be 500–800 J/cm^2^. In our experience in mice tumor models, we see activation of compound **13**^2+^ (Figure 5) at doses around 38 J/cm^2^,^76^ but to our knowledge there is no published paper yet quantifying the necessary light dose for a PACT compound to be activated *in vivo*. In the zebrafish embryo, the whole animal (including eyes) is irradiated, which may generate light toxicity. In a recent PACT study using green light activation (520 nm), our group determined that the maximum tolerated irradiation time under a light intensity of 21 mW/cm^2^ was 6 h, which corresponded to a light dose of 450 J/cm^2^.^64^ After 12 or 24 h irradiation at the same intensity, 50% or 100% dead embryos were observed, respectively. There is hence a limit to the amount of light that a zebrafish embryo can handle. Here as well, at much lower light doses we saw clear activation for **5**^2+^ (Figure 5) in an orthotopic model of an eye tumor following four consecutive PACT treatments of 90 min irradiation time (114 J/cm^2^) each. For this compound, the photosubstitution quantum yield in deaerated acetonitrile was 0.11 (Table 1). Overall, our current experience *in vivo* is that light is usually not a problem for activating PACT compounds with photosubstitution quantum yields of a few percent. However, more *in vivo* data are needed to determine the link between the photosubstitution quantum yield of Ru-based PACT compounds, measured with chemical methods in aqueous or acetonitrile conditions, and *in vivo* activation of their antitumor properties.

### 2.4 Toward Red or Near-Infrared Light Activation

**Figure 6:**
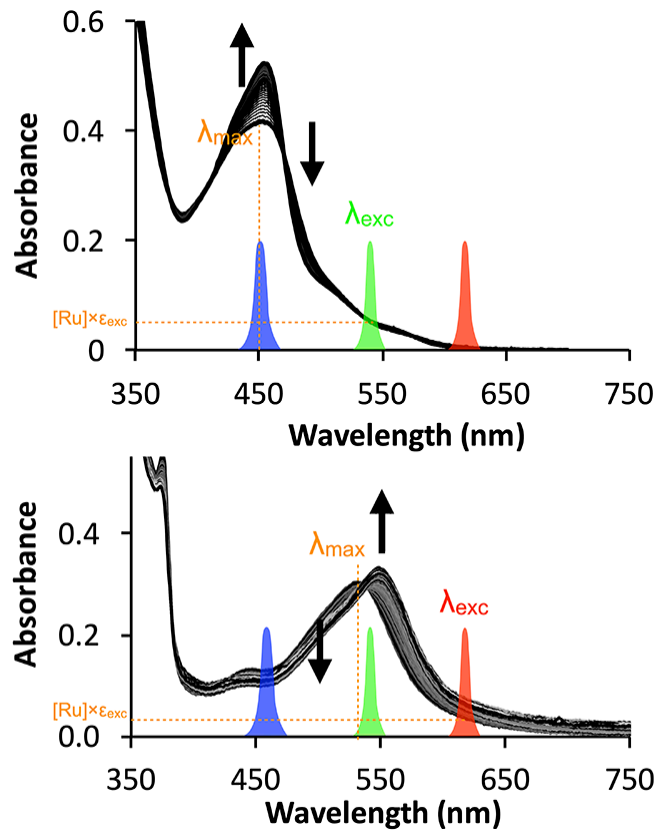
For ruthenium-based PACT compounds, the activation wavelength λ_exc_ does not have to coincide with the absorption maximum λ_max_. Top: **13**^2+^ (see Figure 5) has a maximum in the blue (λ_max_ = 452 nm) but was activated with green light (λ_exc_ = 520 nm, ε_exc_ = 1510 M^–1^·cm^–1^) *in vitro* and *in vivo*. Red light (630 nm) hardly activated the compound. Image developed using data from ref (76). Copyright 2019 American Chemical Society. Bottom: **12**^2+^ (see Figure 5) has λ_max_ shifted to the green (531 nm), which allowed red light activation (λ_exc_ = 625 nm, ε_exc_ = 379 M^–1^·cm^–1^). Image developed using data from ref (78). Copyright 2017 Wiley-VCH.

In phototherapy, the wavelength necessary for triggering light activation is very important. It should be part of the so-called first PDT window (600–1000 nm), a region of the spectrum where light penetrates optimally (up to 1 cm) in biological tissues.^97^ Most ruthenium polypyridyl complexes have their lowest-energy absorption maximum in the blue or green region of the spectrum, which is perfect *in vitro* but suboptimal *in vivo*. Only a few PACT complexes were demonstrated to be photoactivated using red or near-infrared light, though the library of available compound is increasing steadily.^92^ It should be noted that it has remained impossible to shift the lowest energy ^1^MLCT absorption maximum itself, λ_max_ (Figure 6), to the red of the NIR region of the spectrum. A preferred strategy is to bathochromically shift this absorption maximum as much as possible, which increases ε_exc_, the molar absorption coefficient of the compound at the excitation wavelength λ_exc_ (Figure 6). One can then activate the complex with excitation wavelengths λ_exc_ that are red-shifted compared to the absorption maximum λ_max_ of the compound, and located in the red or NIR region of the spectrum. φ_PS_ poorly depends indeed on the excitation wavelength. This observation is in line with Vavilov’s rule, which states that the fluorescence spectrum and quantum yield of a fluorophore are independent of the excitation wavelength. For photoreactivities based on triplet states, the topology of the excited-state hypersurface may be more complex than for a fluorophore, but the few photosubstitutionally active compounds for which φ_PS_ has been measured at different wavelengths usually confirmed this principle. For example, for **13**^2+^ (Figure 5), φ_PS_ was 0.0055 with blue light and 0.0038 with green light,^76^ and [Ru(tpy)(MeCN)_3_]^2+^ photosubstitutes one of the axial MeCN by chloride at 298 K, with a quantum yield φ_PS_ = 0.040 at 436 nm and 0.041 at 480 nm in CH_2_Cl_2_.^88^ Overall, provided that the molar absorption coefficient at the excitation wavelength is not zero, it is possible to excite a PACT compound on the right edge of its main absorption band without paying too much penalty on φ_PS_; one only needs to provide enough photons.

**Figure 7:**
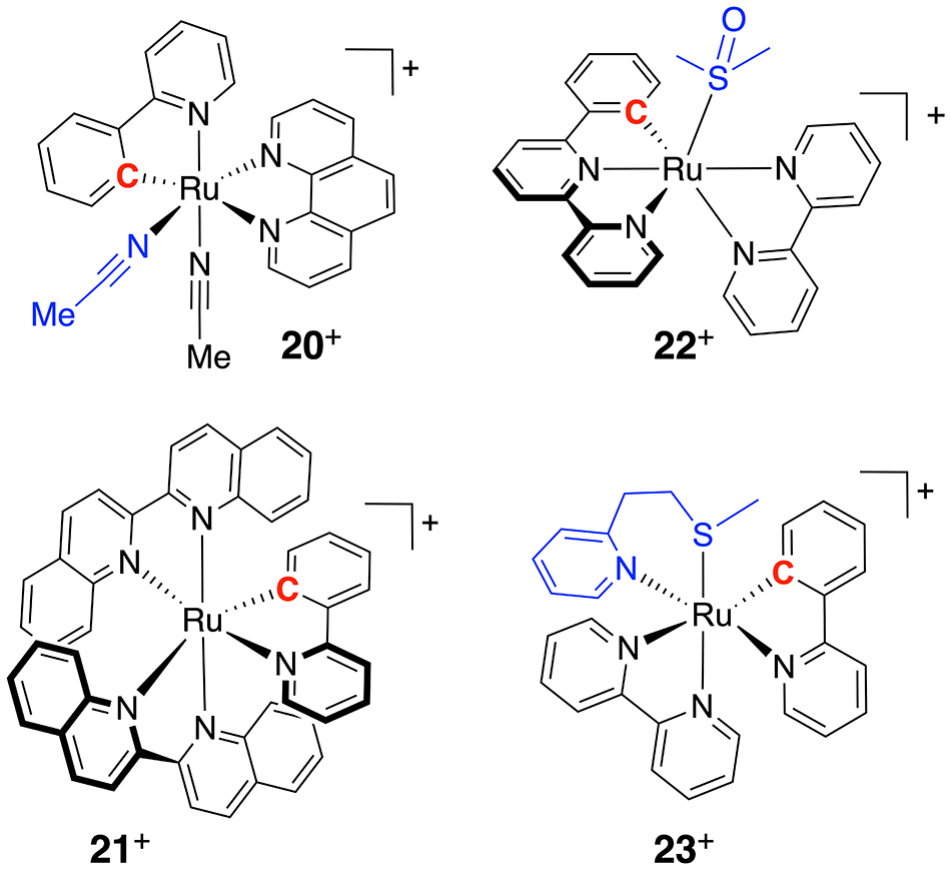
Examples of cyclometalated complexes investigated in Ru-based PACT. The ligand that is photosubstituted first is colored in blue and the carbon atom bound to ruthenium in red.

One classical strategy to implement a red shift in the absorption maximum of a polypyridyl compound is to prepare a “cyclometalated” analogue, in which one of the metal–pyridyl bonds is replaced by a metal–phenylene bond (Figure 7). As phenylene ligands are π-donors while polypyridyl ligands are π-acceptors, cyclometalated compounds have higher t_2g_ orbitals, which shifts their MLCT states toward lower energies, and hence their absorption bands to higher wavelengths. Red or NIR light activation was obtained, for example, at 690 nm for the aquation of [Ru(phpy)(phen)(MeCN)_2_]^+^ (**20**^+^, phen = 1,10-phenanthroline, Hphpy = 2-phenylpyridine).^98^ Cyclometalated complexes have also a lower charge, which helps them penetrate cell membranes. They can hence be excellent anticancer drugs, PDT sensitizers, or protein inhibitors. On the other hand, cyclometalation is often detrimental for the photosubstitution efficacy of bidentate chelates;^99^ for example, [Ru(phpy)(biq)_2_]^+^ (**21**^+^, biq = 2,2′-biquinoline) is completely photoinert. The negative charge borne by phenylene increases the ligand field splitting energy of the complex, which increases the ^3^MC energy and Δ*G*_0_ (Figure 3a) and lowers φ_PS_. To keep the dissociative ^3^MC states low enough for photosubstitution to occur, the coordination octahedron should be distorted. Our group demonstrated this principle for the cyclometalated complex [Ru(phbpy)(bpy)(dmso)]^+^ (**22**^+^, Hphbpy = 6-phenyl-2,2′-bipyridine), in which the terpyridine effect generated enough distortion in the coordination octahedron to allow photosubstitution of dmso by acetonitrile. The quantum yield of this reaction was low, however (0.00041 at 450 nm, compared to 0.016 for its terpyridine analogue).^74^ Steric hindrance could also be introduced in the form of a six-membered metallacycle obtained by coordinating the N,S chelate 2-(methylthio)ethyl-2-pyridine (mtep) to obtain the heteroleptic complexes [Ru(bpy)(phpy)(mtep)]^+^ (**23**^+^).^68^ Mtep photosubstitution worked in acetonitrile (φ_PS_ = 0.00035 at 521 nm) but was 1 order of magnitude slower than for the bipyridyl analogue [Ru(bpy)_2_(mtep)]^2+^ (φ_PS_ = 0.0030). These compounds, together with **20**^+^, belong to the few reported cyclometalated complexes capable of photosubstitution. Their biological properties remain unknown, however, and in general the cyclometalation strategy has not been shown (yet) to lead to efficient PACT compounds.

As discussed above, a more successful strategy to bathochromically shift the absorbance maximum of ruthenium polypyridyl compounds and obtain light activation with red or NIR light was introduced by Turro.^51^ It consisted of changing bpy in [Ru(tpy)(bpy)(L)]^2+^ into an oxygen-based acac^–^ chelate to obtain [Ru(tpy)(acac)(RCN)]^+^ complexes (**1**^+^, Figure 5). By doing so, the π-accepting bipyridyl ligands are replaced by a σ-donor chelate with weak π-donor properties, which comparatively increases the energy of the t_2g_ orbitals. The resulting ^3^MLCT states are dramatically lowered in energy, compared to bipyridine analogues, which shifts absorption toward the NIR region of the spectrum. This strategy was recently extended by the Glazer and Sun groups, who provided phototoxic compounds biologically activated by NIR light.^100,92^ In contrast to [Ru(tpy)(acac)(MeCN)]^+^, with Glazer’s [Ru(bpy)_2_(acac)]^+^ and analogues, there was no reported sign of photosubstitution, and the compound worked via a PDT mechanism. This observation fits with the classical mechanism of photosubstitution, where low ^3^MLCT states and lack of distortion of the coordination sphere are detrimental for the thermal promotion of ^3^MLCT states to ^3^MC states. It is unclear at this stage why photosubstitution from ^3^MLCT states would work with terpyridine-based complexes [Ru(tpy)(O-O)(L)]^+^ and not with bis-bipyridine complexes [Ru(bpy)_2_(O-O)]^+^. It should be noted, however, that similar deviations from the classical model of photosubstitution have also been observed by Etchenique’s groups. Although *trans*-**9**^2+^ has a red-shifted absorption maximum (464 nm) compared to *cis*-**9**^2+^ (432 nm), and hence a lower ^1^MLCT state, it also has a higher photosubstitution quantum yield (Table 1).

### 2.5 Photocaging: A Working Strategy ... with a Twist

In principle, in PACT the photocaged compound cannot inhibit its targeted protein at all, nor bind to DNA. This is a simple idea, but a great majority of the work discussed in this Perspective has effectively shown experimentally that the photocaged inhibitor was (much) less active in the dark than after light activation. For example, compounds **13**^2+^ and **15**, shown in Figure 5, really cannot inhibit tubulin polymerization and CYP1B1, respectively. Other works, for example, from Etchenique on neurotransmitters,^101^ Turro on cathepsin inhibitors,^102^ Glazer on P450 inhibitors,^103^ or more recently Zhang on the kinase inhibitor sorafenib,^104^ have demonstrated similarly that the ruthenium caging groups do their caging job properly.

**Figure 8:**
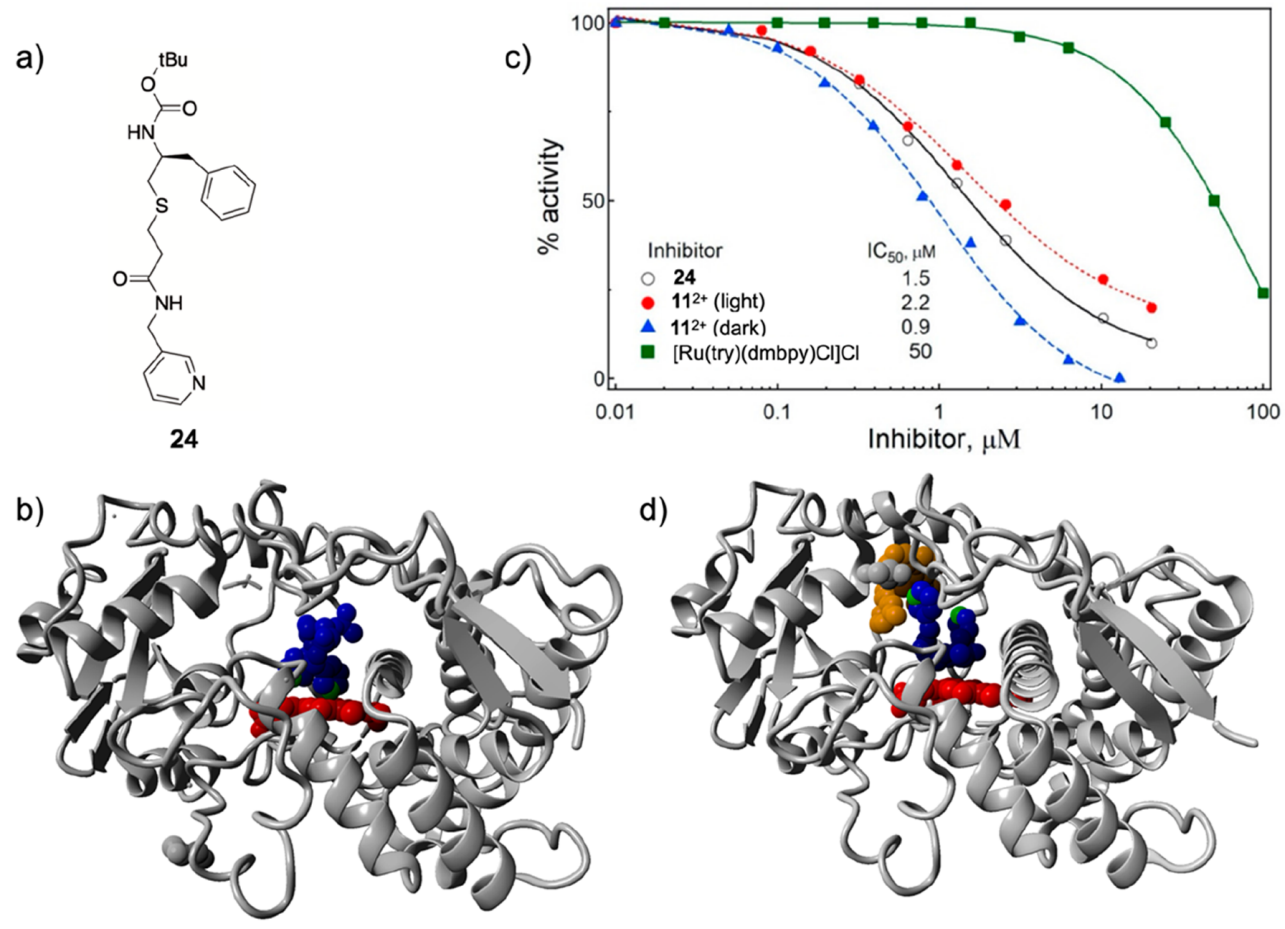
(a) Formula of the CYP3A4-inhibiting ritonavir analogue **24** photocaged by Turro et al. with the [Ru(tpy)(dmbpy)]^2+^ moiety. (b) X-ray structure of the uncaged CYP3A4 inhibitor shown in (a) bound via pyridine coordination to the heme iron center (PDB: 4D78). (c) Protein activity dose–response curves for the uncaged inhibitor **24**, the ruthenium-caged inhibitor **11**^2+^ (Figure 5) in the dark and after light activation, and the control ruthenium caging group [Ru(tpy)(dmbpy)Cl]Cl. (d) X-ray structure of the caged CYP3A4 inhibitor **11**^2+^ interacting with CYP3A4 without pyridine coordination to the heme iron center (PDB: 7KS8). Color code: pyridine nitrogen atoms are in green, heme is in red, the inhibitor **24** is in blue, and the ruthenium caging group of **11**^2+^ is in orange. Adapted from ref (80). Copyright 2021 American Chemical Society.

In some cases, however, the dark toxicity of non-activated ruthenium-based PACT prodrugs, or the protein inhibition properties of the caged compound in the dark, were reported to be significant.^78^ Recently, the Turro and Kodanko groups found out an explanation for this observation: their caged molecule **11**^2+^ (Figure 5) was found to be a better inhibitor of the major human drug-metabolizing enzyme CYP3A4 than the uncaged ligand **24** (Figure 8a).^80^ CYP3A4 belongs to the large P450 family of heme proteins capable of oxidizing hydrophobic drugs to increase their water solubility. Classical CYP3A4 inhibitors such as **24** contain a coordinating pyridine ligand which binds to the iron heme center, thereby blocking the catalytic center (Figure 8b). The ruthenium caged inhibitor **11**^2+^ was shown via an enzyme activity assay (Figure 8c) and an X-ray structure of the protein (Figure 8d) to better fill the protein binding pocket than the uncaged inhibitor itself. Of course, in the caged compound **11**^2+^, the pyridine ligand of the inhibitor **24** is engaged in coordination to ruthenium, so it cannot bind to heme, but the shape of the ruthenium prodrug turned out to be ideal for filling the catalytic pocket and preventing substrates from reaching the catalytic center. At this stage, this unexpected effect was clearly demonstrated in only one case. However, it is probable that it may play a role in other ruthenium-based PACT compounds as well. As an example, when the NAMPT inhibitor called STF31 (IC_50_ = 0.25 μM) was caged into compound [Ru(tpy)(biq)(STF31)]^2+^ (**12**^2+^ in Figure 5), it became 18 times less potent (IC_50,dark_ = 4.8 μM).^78^ Such a caging effect is significant and corresponds to expectations. On the other hand, the IC_50_ value for NAMPT inhibition by **12**^2+^ in the dark was not negligible, which suggested some form of interaction between the Ru-caged prodrug and the protein. Like for **11**^2+^, the enzyme inhibition properties of **12**^2+^ in the dark may also explain, at least in part, the significant dark toxicity of this compound toward cancer cells (e.g., EC_50,dark_ = 23.6 μM in A431 skin cancer cells in normoxia). Although similar unwanted inhibitory properties are observed every now and then with metal complexes,^105^ it should be noted that they are more an exception than the rule. In addition, it should be possible to correct them by changing the ruthenium cage or the place where it is installed on the inhibitor, to lower interaction with the targeted protein. Overall, the principle of photocaging, where installing the ruthenium photocage suppresses the biological properties of an inhibitor, is a simple and working idea.

### 2.6 What Do We Need to Optimize?

**Figure 9:**
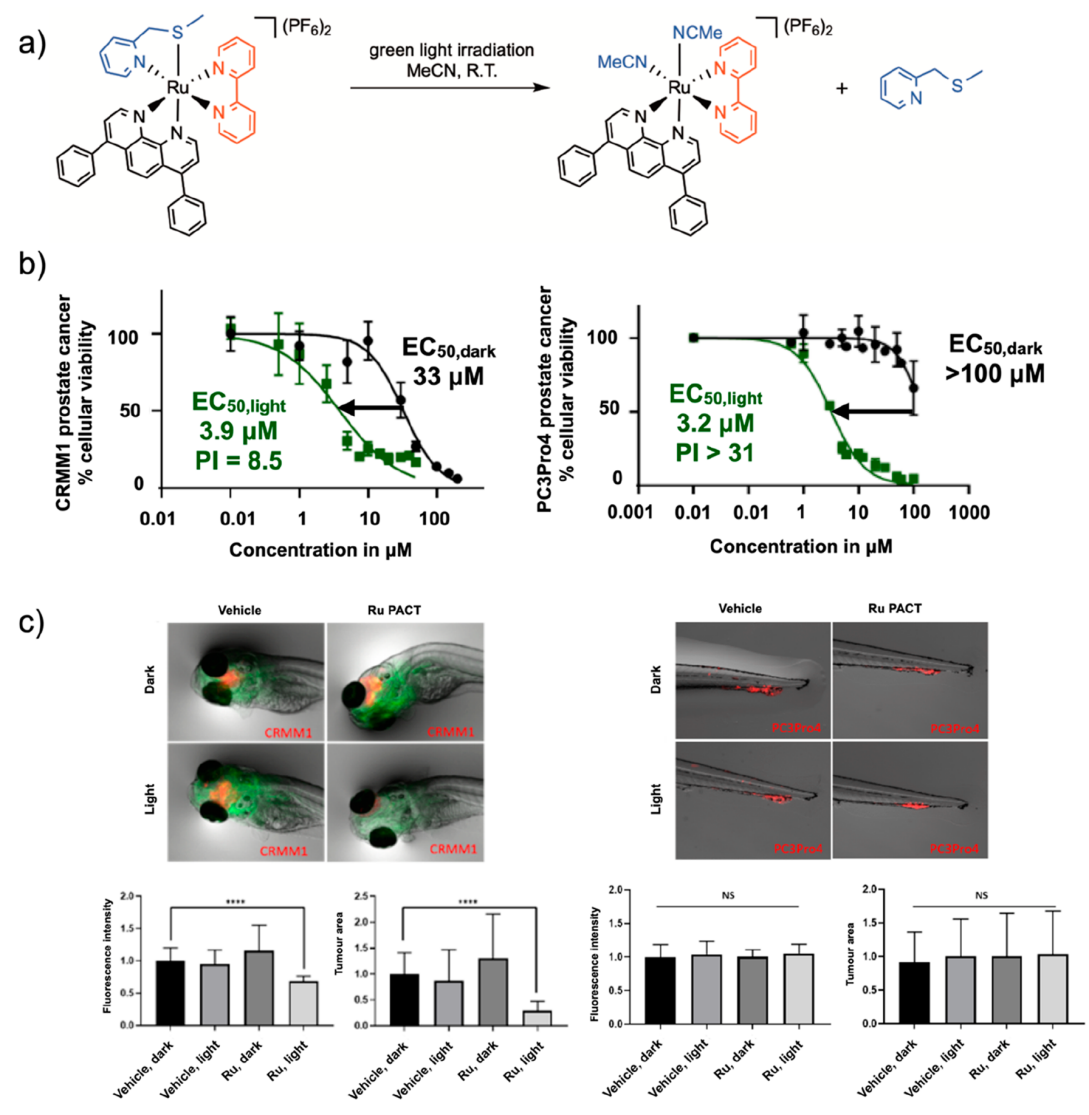
Non-trivial relationship between *in vitro* and *in vivo* performances of the PACT compound [**5**](PF_6_)_2_. (a) Photosubstitution reaction for **5**^2+^ irradiated with green light in acetonitrile. (b) *In vitro* dose–response curves for **5**^2+^ in CRMM1 eye cancer cell (PI ≈ 8.5) and PC3Pro4 prostate cancer cells (PI > 31). (c) *In vivo* performance of **5**^2+^ under green light activation (520 nm, 114 J/cm^2^) in an orthotopic CRMM1 eye tumor model in zebrafish embryo (left) and in a PC3Pro4 ectopic prostate zebrafish tumor model (right). Green fluorescence shows blood vessels, and red fluorescence shows the tumor cells. ****p < 0.0001. Reproduced with permission from ref (64). Copyright 2022 Royal Society of Chemistry.

In most PACT studies, researchers tend to maximize the photoindex (PI) value of their compounds *in vitro*. Although this idea seems reasonable, the relationship between the PI value *in vitro* and the light-activated antitumor activity *in vivo* is, however, not straightforward to establish. In fact, there are many molecular parameters other than the PI value that need to be optimized to obtain good ruthenium-based compounds for *in vivo* PACT tumor treatment. In a recent collaboration with the Snaar-Jagalska group,^64^ our group studied in zebrafish embryo tumor models the antitumor activity of **5**^2+^ under green light activation (Figure 9). *In vitro*, the PI value of this compound in PC3Pro4 prostate cancer cell lines was >31, which is much better than the PI value found in conjunctival melanoma cancer cell lines CRMM1 and CRMM2 (8.5 and 8.8, respectively). However, *in vivo*, we observed no antitumor activity in the prostate tumor model used, while good antitumor activity was observed in the eye tumor model. An important difference in the type of tumor models should be highlighted here. In the prostate cancer model used, which is called “ectopic”, the human prostate cells were injected intravenously and settled as a tumor in the tail fin of the embryo, which does not correspond to the (prostate) tissue from which the cells originate. The PACT compound was administered either in the water in which the embryo swum, which would require compound uptake through the skin and/or the bronchia, or by intravenous injection. In both cases, no antitumor effect was observed, despite the excellent *in vitro* properties of this compound. Probably, **5**^2+^ never reached the tumor or reached it in too small quantities. By contrast, in the eye cancer model, which is called “orthotopic”, the retro-orbital tumor was installed by injection of the cancer cells behind the eye, which corresponds to the origin of the tumor cells. The compound was also injected retro-orbitally, which led to a clear antitumor effect (Figure 9). Clearly, access of the prodrug to the tumor worked better in the second case, despite the lower PI value *in vitro*. These results highlight that drug delivery is critical *in vivo*. Though they cannot be modeled easily *in vitro*, drug delivery aspects must be considered as well when designing new ruthenium-based PACT compounds. It should also be highlighted that it will be impossible to progress in the field of ruthenium-based PACT without more *in vivo* data.

### 2.7 General Considerations on the Design of Ru-Based PACT Compounds

**Figure 10:**
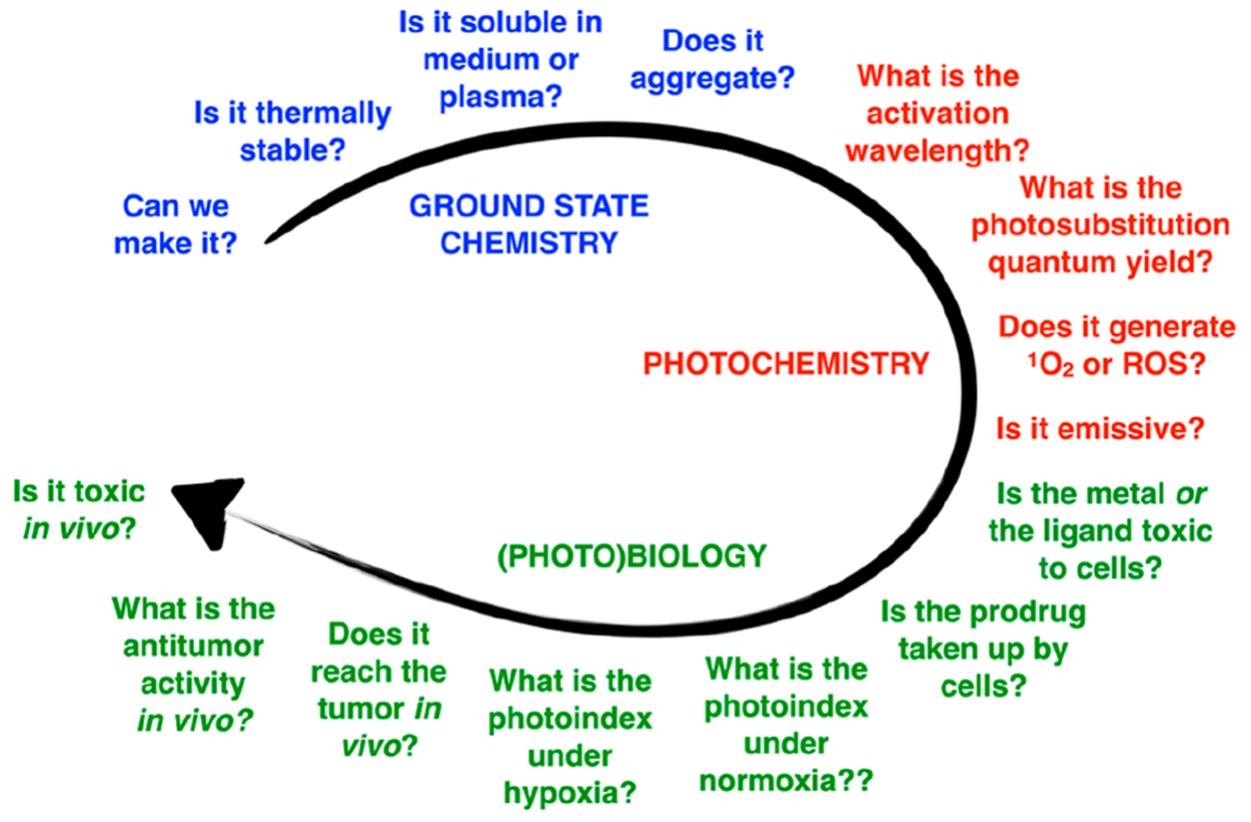
General design aspects for the ruthenium-based PACT compound.

Overall, the design of ruthenium-based PACT compounds must address several issues altogether (Figure 10). Some of the questions regard the ground-state chemical properties of the molecule, such as its synthetic availability, solubility in water (log *P*), aggregation properties, and dark stability. The next set of questions concern the photochemical properties of the molecules: its absorbance spectrum, whether it absorbs red or NIR light, the photosubstitution quantum efficiency at different wavelengths, as well as the quantum efficiency of ^1^O_2_ generation. Last but not least, the biological properties are critical: whether the metal- or ligand-based photoproduct generates phototoxicity,^63^ whether or not the molecule targets the tumor, whether and how well the compound is taken up by cancer cells in the non-activated form or in the activated form, whether the molecule is photoactivated under normoxia, whether it also works under hypoxia, whether it works *in vivo*, and how toxic it is to the animal, in particular to the kidneys and liver. PACT represents an advanced form of traditional chemotherapy, and the number of properties to be optimized altogether makes the clinical development of PACT a challenging process.

## 3 Biological and Clinical Considerations of Ru-Based PACT

### 3.1 Which Clinical Application for PACT?

In principle, PACT is a very general technology, as a large variety of protein inhibitors can be photocaged with a large variety of ruthenium complexes. On the other hand, Ru-based PACT is still in its infancy as it is not yet applied in clinics. In fact, its potential for real-life applications relies on the immense experience of PDT oncologists throughout the world who have performed phototherapeutic cancer treatment using clinically approved sensitizers such as Photofrin (skin, esophagus), 5-ALA (glioblastoma), mTHPC (head-and-neck), Visudyne (eye), or Padeliporfin (prostate).^8^ According to clinicians using PDT, for many tumors, surgical removal is preferred to phototherapeutic treatment. However, specific types of tumors are more relevant for phototherapeutic treatment than for surgery. First, in patients with multiple small tumors, such as basal cell carcinomas (BCCs) on the back, surgical removal can be problematic, and PDT is often preferred.^106^ In general, early tumors represent an interesting field of application for phototherapy, as a cream application or intravenous injection, followed by light irradiation, is usually simpler than surgery. For example, retinoblastoma in the eyes of infants can be treated by PDT with fewer side-effects than surgery,^107^ and Barrett’s esophagus has been one of the main clinical applications of PDT since 1994. Second, PDT is also used for tumors where surgery is too debilitating, such as for patients with non-resectable brain tumors,^108^ Paget’s disease of the vulva,^109^ sinus tumors,^110^ or tongue tumors.^111^ Last but not least, PDT is currently being developed in clinics as an adjuvant treatment to surgery, notably to prevent recurrences. For example, clinical trials are currently undergoing for brain tumors (NCT05363826), lung cancer (NCT02662504), or Paget’s disease.^112^ The idea in this strategy is to insert the phototherapeutic treatment in the standard-of-care procedure with minimal discomfort and minimal safety issues for the patient, while disease control is improved. Altogether, the currently used or new applications of PDT should be taken as inspiration for the future development of Ru-based PACT in the clinics.

**Figure 11:**
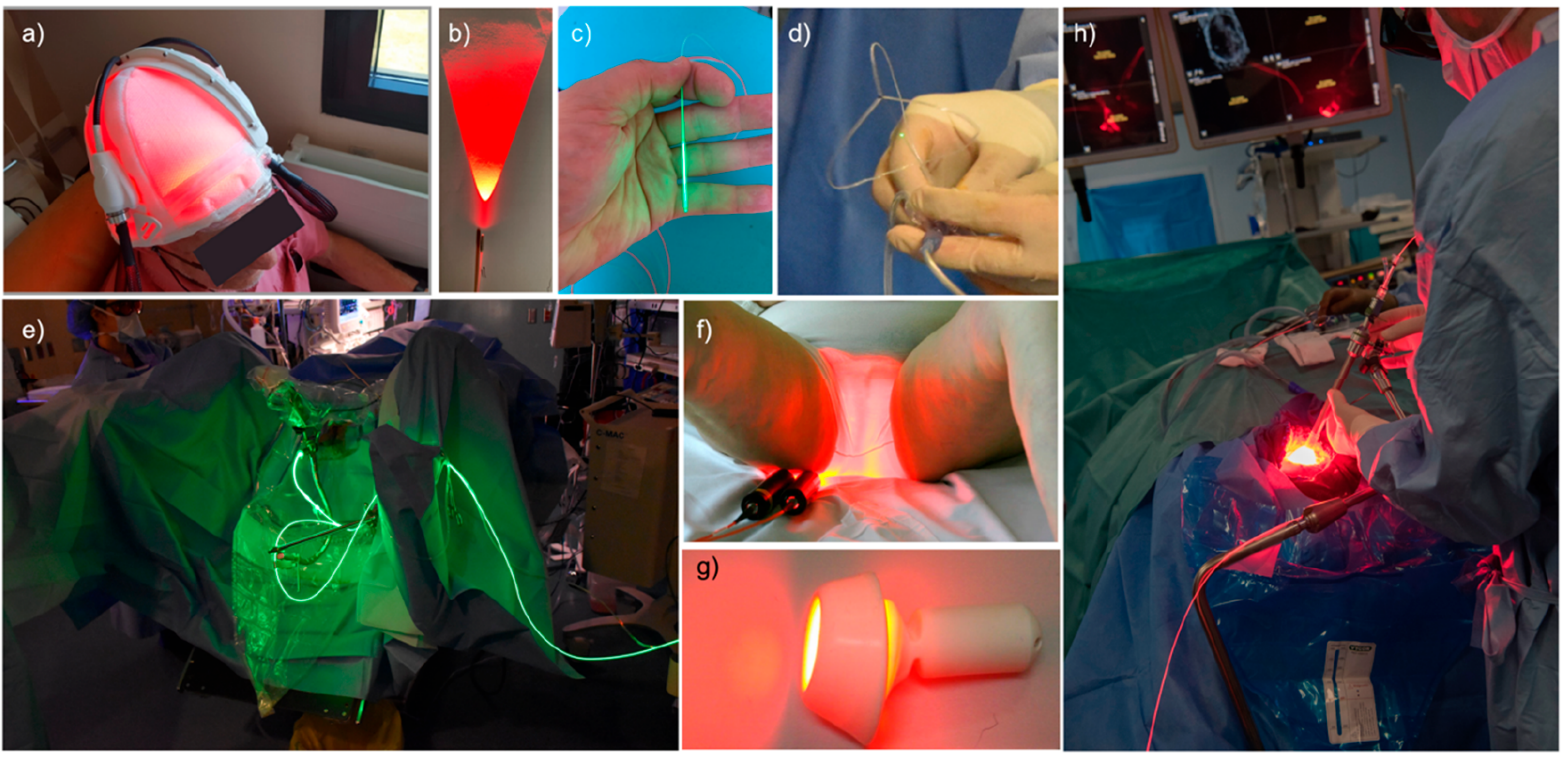
Lasers and devices used to shine light in patients for anticancer phototherapy. (a) Fabric-based biophotonic device used for Phosistos photodynamic therapy of actinic keratosis.^113^ (b) Frontal light delivery using an optical fiber, for example, for irradiation of skin tumors. (c) Radial light distribution for interstitial photodynamic therapy using an optical fiber terminated by a light diffuser. (d) The TLC-3200 system for simultaneous green light irradiation of bladder tumors (532 nm) and light dosimetry during PDT treatment with TLD-1433.^114^ (e) Clinical setup of PDT bladder cancer treatment using TLD-1433 and green light. Image courtesy Lothar Lilge. (f) PAGETEX device for controlled vulvar illumination with 635 nm light in the PDT treatment of primary extramammary Paget’s disease of the vulva. Reprinted with permission under a Creative Commons CC BY 4.0 from ref (109). Copyright 2020 The Authors, published by Wiley-VCH. (g) Cevira device for cervix illumination with light. Image courtesy Serge Mordon. (h) Homogeneous light diffusion with a light-scattering balloon inflated in the excised primary tumor cavity for intraoperative PDT treatment of glioblastoma (INDYGO trial). Reprinted with permission under a Creative Commons CC BY 4.0 from ref (115). Copyright 2020 The Authors, published by Springer.

As noted, there have been intense clinical efforts toward the industrial development of medical lasers and light-irradiation devices to bring light into the human body. Therefore, it is nowadays possible to shine light on most parts of the body. A few representative examples of light-irradiation devices are shown in Figure 11. Because of these developments, PACT researchers do not need to develop new light delivery techniques for clinical applications. They could use existing devices and techniques for the development of PACT compounds. On the other hand, the development of new light-irradiation devices represents an interesting avenue for the industrial development of new, integrated phototherapeutic solutions for cancer treatment. Such products may contain both the PACT compound itself, appropriately formulated, and a device to shine light in an appropriate manner (shape, wavelength, light intensity, protocol) on the organ targeted by the compound. These matters should be taken seriously by PACT researchers, considering the time and money needed for developing any new molecule toward the market.

### 3.2 Hypoxia in Oncology

PDT is extremely efficient in a range of diseases when surgery is either impossible (some forms of liver metastases, pancreas tumors, etc.) or strongly debilitating (brain, genitals, face, etc.). PDT is also ideally suited for treating cancer in developing countries, where access to last-generation chemotherapy, antibiotics, or radiation therapy equipment is insufficient.^116^ Ideally, Ru-based PACT should not compete with PDT but bring new solutions where PDT is not working. An essential question in the field of PACT is which application is the most promising for this new technology, and for which disease PACT represents a solution that the clinically more advanced techniques (i.e., PDT) cannot address efficiently. If this question can be answered, PACT has a chance to reach the clinics.

**Figure 12:**
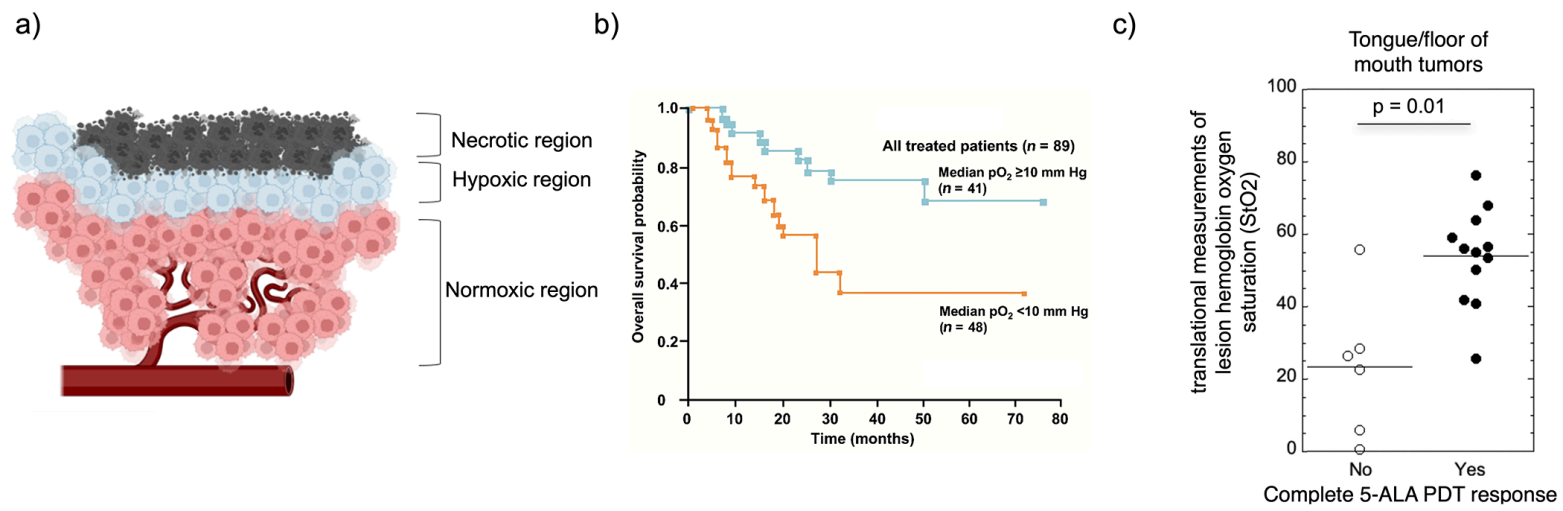
Hypoxia in oncology. (a) Three regions around a blood vessel in a tumor. Image courtesy Iris Kort. (b) Association between tumor hypoxia and overall survival in advanced cancer of the uterine cervix. Reprinted with permission from Vaupel et al., Association between Tumor Hypoxia and Malignant Progression in Advanced Cancer of the Uterine Cervix. *Cancer Res.*, **1996**, *56*, 4509–4515. Copyright 1996 American Association for Cancer Research. (c) Clinical response to 5-ALA PDT treatment in tongue/floor of mouth tumor patients. Reproduced with permission from Busch et al., Lesion oxygenation associates with clinical outcomes in premalignant and early stage head and neck tumors treated on a phase 1 trial of photodynamic therapy. *Photodiagnosis Photodynamic Therapy***2018**, *21*, 28–35. Copyright 2018 Elsevier.

Following this approach, our group introduced the idea that the non-dependence of photosubstitution reactions on molecular oxygen may make PACT ideal for the treatment of hypoxic tumors,^78^ which are difficult to treat with approved PDT sensitizers.^117^ Hypoxia is qualitatively defined as an abnormally low concentration of molecular oxygen in biological tissue. It can be a state of disease, but it also occurs as part of the natural evolution of embryo development or tissue regeneration in healthy individuals. Hypoxia occurs whenever the number of cells around a blood vessel becomes too large for available O_2_ delivery (Figure 12a). Cells might develop too quickly for the available blood supply, such as in an embryo or in a tumor, but in other cases, the blood supply may become suddenly or chronically impaired because of a wound, of PDT treatment, of ischemia, of high altitudes, or of apnea.^118^ As hypoxia is a natural phenomenon, cells have evolved different mechanisms to cope with it, which are primarily controlled by the transcription factors HIF1α and HIF2α.^119^ These mechanisms are highjacked in cancer cells,^120^ which must always face an abnormally low state of oxygenation during early tumor development. Following HIF1α activation, hypoxia leads to the overexpression of different factors, such as the vascular endothelial growth factor (VEGF) to grow new blood vessels, carbonic anhydrase,^121^ the glucose receptor (GLUT1), and different genes related to the reprogramming of cell metabolism toward glycolysis, a phenomenon called the Warburg effect. Hypoxia also pushes cancer cells to seek for better oxygenated tissues, which drives the metastasis cascade: the cells undergo the epithelial–mesenchymal transition (EMT), escape the primary tumor into the bloodstream, and re-settle in a distant tissue where they start to grow again and generate more blood vessels.

Clinically speaking, it is possible to measure, upon cancer diagnosis, the percentage of the tumor volume that shows low O_2_ concentration.^122^ This percentage has been shown to be positively correlated to the efficiency of anticancer therapies: the less O_2_ present in the tumor tissue at diagnosis, the lower the patient’s chance of survival, all treatment considered (see an example for cervical cancer in Figure 12b).^45,46^ Importantly, this correlation is not only observed for PDT, where a lack of dioxygen obviously leads to less ROS and hence less antitumor effect (Figure 12c), but it also holds for radiation therapy,^47^ immunotherapy,^48^ or chemotherapy.^49^*Solving the hypoxia problem is hence an important issue in oncology*,^50^ and we are convinced that developing Ru-based PACT specifically to address this problem can be a clinically relevant approach.

### 3.3 Ruthenium-Based PACT for the Treatment of Hypoxic Tumors

In fact, hypoxia is understood differently by chemists and biologists. Chemically speaking, hypoxia represents a lower O_2_ concentration (typically 1% O_2_) compared to that present in “normoxic” incubators (21% O_2_). In principle, such low O_2_ concentrations lower, at a given excited-state photosensitizer concentration, the rate of ^1^O_2_ formation^123^ and hence the efficacy of PDT. This effect has been reported multiple times for PDT type II photosensitizers,^124,125^ but things get more complicated for PDT type I, which is notoriously less sensitive to hypoxia.^126−128^ Recently, studies by the McFarland and Glazer groups have confirmed these trends for ruthenium-based PDT sensitizers, which in spite of an unknown but ^1^O_2_-independent mechanism behave extremely well under hypoxia.^94,129,130^ Usually, it is argued that PDT type I involves photoredox chemistry: electron transfer from the excited state of the photosensitizer occurs to biomolecules other than O_2_ because the excited states of ruthenium-based sensitizers are particularly good oxidizing *and* reducing agents. A photoreduced sensitizer may further transfer its strongly reductive electron to H_2_O_2_ to produce hydroxy radicals OH^•^ without the involvement of molecular oxygen, which explains the comparatively low sensitivity of PDT type I to hypoxia. In PACT, the photosubstitution reaction quenches the triplet states responsible for ^1^O_2_ formation (usually the ^3^MLCT, ^3^π–π*, or ^3^ILCT states), which makes most PACT compounds too short-lived to be good sensitizers for PDT type II.^78,53^ As a consequence, photosubstitution reactions are typically not efficiently quenched by O_2_, so that they remain more or less effective under hypoxia as under normoxia.^69^

Biologically speaking, however, hypoxia represents something else: essentially a tougher environment for cancer cells. Surviving hypoxia during tumor development selects the most resistant cells, which become biologically different compared to cells that always lived in a normoxic area. Hypoxic cells import and burn glucose via different pathways,^131^ they express many proteins differently, and their sensitivity to apoptosis^132^ or other forms of cell death^133^ is very different. Therefore, even if the photosubstitution reaction in a PACT compound is as efficient under hypoxia as under normoxia, the killing of a cancer cell by the chemical action of a photoreleased cytotoxic molecule may be very dependent on local O_2_ concentrations.

Experimentally speaking, two observations are typically made for most ruthenium-based PACT compounds. On one hand, the PI value remains identical or very similar between normoxia (21% O_2_) and hypoxia (1% O_2_). For example, **12**^2+^ (Figure 5) showed a PI value of 3.6 in hypoxic A431 skin cancer cells (1% O_2_) vs 3.3 in normoxia (21% O_2_).^78^ Other compounds, such as **13**^2+^ (Figure 5), showed a PI of 4.0 vs 4.1 in normoxic vs hypoxic A549 cells, respectively.^76^ On the other hand, the cell growth inhibition effective concentration (EC_50_) values in the dark and under light activation of these compounds became higher under hypoxia, which means that PACT molecules become less toxic in hypoxic cells. For **13**^2+^ the identical PI values obtained at 21% and 1% O_2_ were in fact obtained from different EC_50,dark_ and EC_50,light_ values: 35 and 9.2 μM in normoxia vs 55 and 14 μM in hypoxia, respectively. Thus, though it is possible to affirm that the *activation* of a ruthenium-based PACT compound is independent of the O_2_ concentration, it would be incorrect to say that the *phototoxicity* of a ruthenium-based PACT compound does not depend on the O_2_ concentration.

In fact, chemically speaking, the distinction of a PDT vs PACT compound is easy to establish by measuring the quantum yields of photosubstitution and of ^1^O_2_ generation (Table 1): PDT compounds have high Φ_Δ_ (>0.20) and low Φ_PS_ (<0.001) values, whereas PACT molecules have a low Φ_Δ_ (<0.10) and a high Φ_PS_ (>0.001). However, making a clear-cut experimental distinction between PDT and PACT in biological conditions is much more difficult, in particular for compounds such as **16**^2+^ (Figure 5) that can do both.^77,134,94^*In vitro*, for PDT compounds the lower efficacy of light-induced cell killing at low O_2_ concentrations, compared to normoxia, leads to a dramatically lower photoindex under hypoxic conditions. This observation is usually *interpreted* as a chemical consequence of the low O_2_ concentration—at least for PDT type II compounds. For PACT compounds, light-induced cell killing under hypoxic conditions is also often less efficient as EC_50,light_ becomes higher, but the *interpretation* of this observation is usually different: the cells have, biologically speaking, become more resistant to chemotherapy due to the activation of the hypoxia response of the cell—not due to the chemical unavailability of ^3^O_2_. Experimentally speaking, a difference between the two effects can be made, as the PI values of typical PACT compounds remain similar at low vs high O_2_ concentration, while the PI values of PDT compounds usually become much lower in hypoxia. It is also possible to experimentally observe another difference between PDT and PACT: intracellular ROS generation is negligible for PACT compounds, while it is strong for PDT compounds. In fact, a similar PI value in normoxic and hypoxic conditions *in vitro* may be considered a hallmark of PACT compounds, while PI values that vary with the O_2_ concentration may be signs of a cell-killing mechanism involving a photodynamic effect. However, these effects were recently shown to be dependent on the activation wavelength,^94^ while little data exists for PDT type I compounds. At this stage, this statement should be only taken as a proposition, and the distinction between PDT and PACT mechanisms in biological conditions remains an open question.

**Figure 13:**
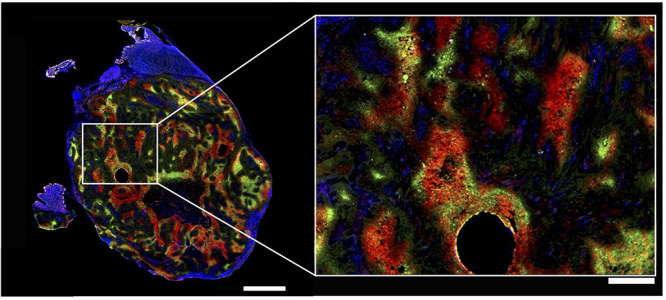
Heterogeneity of hypoxia in hind limb SQ20b human squamous cell carcinoma subcutaneous xenograft in mice shown by three-color hypoxia imaging. Blue is Hoechst 33342 (nuclei), green is pimonidazole (hypoxia marker 1), and red is carbonic anhydrase (hypoxia marker 2). Left bar is 2 mm, right bar is 500 μm. The right image shows high magnification of the region of interest shown on the left by a white rectangle. The circular hole was caused by angiocatheter placement before tumor sectioning. This research was originally published in ref (135). Copyright 2014 Society of Nuclear Medicine and Molecular Imaging, Inc.

*In vivo*, the situation is more complicated. First, each tumor contains both regions of normoxia and regions of different levels of hypoxia (Figure 13). Therefore, for compounds combining PDT and PACT, different modes of cell killing might take place within the same tumor, depending on the local O_2_ concentration. In addition, the difficulty of killing intrinsically more resistant hypoxic cancer cells should not hide the problem of drug penetration into hypoxic regions of a tumor, which is a serious issue both for PDT and PACT compounds. Hypoxic regions in tumors are hypoxic precisely because they are badly vascularized, which prevents not only O_2_ delivery but also drug delivery via blood circulation. Overall, solving the hypoxia problem in phototherapy will require not only developing photocaged compounds that can kill more resistant cells but also looking for innovative drug delivery strategies that can bring such drugs into hypoxic areas. Molecular compounds that diffuse efficiently into hypoxic areas are, in fact, needed here as well. Solving this difficulty will require more *in vivo* studies.

### 3.4 Tissue Necrosis or Apoptosis?

As highlighted above, phototherapy modalities are more promising for two types of clinical issues. The first are non-resectable tumors, for which surgery cannot be considered because the danger for the patient is too high—for example, non-resectable brain tumors or tumors located near essential blood vessels such as the aorta, the portal vein, or the liver. Second, phototherapy may also be useful in cases where the tumor is small or badly located for surgery, for example, on the tongue, the face, the bladder, the colon, or the genitals. Next to the lower quality of life, surgery at a hospital can be costly and lead to serious risk for resistant infection, while a simple injection or local application of a light-activated prodrug followed by visible light irradiation of the tumor might represent attractive therapeutic approaches due to their low side effects, low costs (light sources are comparatively cheap), and low risk for contracting resistant infection. Typical examples can be superficial mouth tumors or suspect nevi in the retina, which are precursors for more life-threatening uveal melanoma tumors.^136^

For both types of applications, tissue conservation may be seen as an essential feature of phototherapy. One of the potential issues in clinical PDT is that efficient treatment typically leads to tissue necrosis. On the one hand, tissue necrosis is good, because it triggers the immune system and generates serious antitumor immunity.^137^ On the other hand, necrosis leads to inflammation and pain, which has been reported by many PDT patients. Pain can be managed by lowering the light intensity (in mW/cm^2^) while increasing irradiation time to keep the light dose (in J/cm^2^) constant.^138−140^ However, if necrosis reaches essential tissues, clinical success of a PDT treatment may be an issue for the patient, notably if PDT is performed on tissues that should be conserved. Such effects have been observed in red-light PDT treatment of the bladder using Photofrin in the 1990s, which destroyed part of the healthy muscle tissues underlying the tumor, which stopped clinical trials.^141^ Photoactivated technologies that trigger other forms of cell death, such as apoptosis, ferroptosis, or immunologic cell death, may lead to tumor eradication without pain and without tissue necrosis, which could benefit the development of Ru-based PACT. Apoptosis may not necessarily generate as much immune response as that generated by PDT, which some see as detrimental to the development of PACT. On the other hand, many metal-based drugs have been shown to trigger significant immune response even in the absence of necrotic cell death.^142^ Overall, the immune aspects of Ru-based PACT have not yet been studied and may require more attention in the future.

## 4 Conclusions and Outlook

Ru-based PACT represents a fantastic opportunity for the development of bioinorganic photochemistry. It is a very general approach for cancer therapy, but it may also be relevant for diseases different from cancer. For example, the Etchenique group focuses on the light-induced delivery of neurotransmitters. Recent photopharmacological studies on, for example, light-activated morphin derivatives that alleviate pain by local remote activation without opioid-related adverse effects^14^ may inspire new opportunities for ruthenium-based photocages. Other fields of applications, such as antibiotics, may benefit from light activation as well whenever side effects of antibacterial treatment are problematic.

From the fundamental point of view, the development of a new ruthenium-based scaffold with improved photosubstitution properties at wavelengths closer to the NIR region of the spectrum is still needed. The very recent development of PACT compounds performing photosubstitution from their ^3^MLCT state, rather than via the classical mechanism involving ^3^MC excited states, is still poorly understood and will need additional (theoretical) studies. New developments in the theoretical description of photosubstitution reactions, and notably on the involvement of solvent molecules, are also needed.^50^ In terms of efficacy, very few PACT compounds show photoindexes higher than 50 *in vitro*, in particular, under hypoxia, while PI values of thousands have been reported for PDT compounds in normoxia. Improved PI values may be obtained by combining a toxic ligand and a toxic ruthenium cage that cancel each other when bound in the dark and kill cells synergistically after uncaging, but the validity of this approach has not yet been addressed convincingly. In general, we lack a thorough understanding of the efficacy of PDT vs PACT compounds in the context of hypoxia. As noted, the question of the link between *in vitro* efficacy (i.e., the PI value) and *in vivo* antitumor properties remains open, mostly due to the lack of animal data. Though most researchers in the field try to maximize the photoindexes of new PACT compounds in 2D and 3D cancer cell cultures, a single study from our group compared the efficacy of a PACT compound in two different tumor models and found no correlation between the PI value *in vitro* and the antitumor efficacy *in vivo*.^64^ More studies are clearly needed on this essential topic, in particular, in mice.

On the impact side, the PACT community may also need to look for more clinical relevance, not only to avoid sterile competition with PDT, a technique that is often very efficient and already in the clinics, but also to avoid the “me too” approach, which is detrimental to realistic technological development. In general, too few *in vivo* preclinical studies have been published for Ru-based PACT compounds, which does not allow for answering essential questions such as the biodistribution and systemic toxicity of ruthenium polypyridyl compounds (pharmacokinetic/pharmacodynamic effect), the link between Ru-based PACT and the immune system, or the necessity of making actively tumor-targeted PACT compounds. We should also name the question of the combination of PACT compounds with approved chemotherapy drugs, which has been addressed, to our knowledge, only once, while it may be the only way to reach the clinics.^80^ In order to move toward randomized clinical trials, it would indeed be necessary to compare a group treated with the new technique (Ru-based PACT) plus the best-available treatment (e.g., chemotherapy) to a control group receiving the best-available treatment only. If one wants to ever see Ru-based PACT compounds in the clinics, more pre-clinical data combining ruthenium compounds and approved chemotherapy will be needed.
